# Rare diseases of epigenetic origin: Challenges and opportunities

**DOI:** 10.3389/fgene.2023.1113086

**Published:** 2023-02-06

**Authors:** Maggie P. Fu, Sarah M. Merrill, Mehul Sharma, William T. Gibson, Stuart E. Turvey, Michael S. Kobor

**Affiliations:** ^1^ Department of Medical Genetics, Faculty of Medicine, University of British Columbia, Vancouver, BC, Canada; ^2^ Centre for Molecular Medicine and Therapeutics, University of British Columbia, Vancouver, BC, Canada; ^3^ BC Children’s Hospital Research Institute, Vancouver, BC, Canada; ^4^ Department of Pediatrics, Faculty of Medicine, BC Children’s Hospital, University of British Columbia, Vancouver, BC, Canada

**Keywords:** epigenetics, rare disease, bioinformatics analysis, DNA methylation, histone modification, chromatin remodeler

## Abstract

Rare diseases (RDs), more than 80% of which have a genetic origin, collectively affect approximately 350 million people worldwide. Progress in next-generation sequencing technology has both greatly accelerated the pace of discovery of novel RDs and provided more accurate means for their diagnosis. RDs that are driven by altered epigenetic regulation with an underlying genetic basis are referred to as rare diseases of epigenetic origin (RDEOs). These diseases pose unique challenges in research, as they often show complex genetic and clinical heterogeneity arising from unknown gene–disease mechanisms. Furthermore, multiple other factors, including cell type and developmental time point, can confound attempts to deconvolute the pathophysiology of these disorders. These challenges are further exacerbated by factors that contribute to epigenetic variability and the difficulty of collecting sufficient participant numbers in human studies. However, new molecular and bioinformatics techniques will provide insight into how these disorders manifest over time. This review highlights recent studies addressing these challenges with innovative solutions. Further research will elucidate the mechanisms of action underlying unique RDEOs and facilitate the discovery of treatments and diagnostic biomarkers for screening, thereby improving health trajectories and clinical outcomes of affected patients.

## 1 Introduction

Rare diseases (RDs) are typically defined by a prevalence threshold of 5–76 cases per 100,000 in the population, with a global average incidence for each disease of 4 per 10,000 (Richter et al., 2015). Although individually rare, RDs are common in aggregate, with more than 10,000 RDs reported to date together affecting roughly 350 million people worldwide (about 4.4% of the population) (Boycott et al., 2019). Historically, however, research and development regarding RDs have been underfunded because of the difficulty of advocacy for small numbers of patients affected by any specific disease (Ekins, 2017). Most RDs are Mendelian disorders, where mutations in a single gene can explain the clinical phenotype (Ekins, 2017; Levy et al., 2022). The recent development of next-generation sequencing techniques has greatly accelerated identification of the genetic origins of RDs (Boycott et al., 2019).

Some RDs of genetic origin are driven by altered epigenetic regulation and are referred to as RDs of epigenetic origin (RDEOs). Epigenetics generally refers to the study of potentially mitotically heritable molecular marks that can perpetuate alternative gene activity states with the same underlying DNA sequence (Henikoff and Greally, 2016; Aristizabal et al., 2020; Carter and Zhao, 2021). These marks are highly relevant in early development, because of their roles in the establishment and maintenance of gene expression profiles that are specific to the functioning of defined cell populations (Feng et al., 2010; Henikoff and Greally, 2016). These gene expression profiles constitute a cellular phenotype that is inseparable from its identity; cells are not static entities, but are defined by their function. Therefore, RDEOs typically affect patients from an early age and drive significantly altered cellular functions across multiple systems (Rodenhiser, 2006; Velasco and Francastel, 2019; Janssen and Lorincz, 2022). RDEOs therefore often present as a combination of immunological, neurological, and physical developmental disorders, perhaps because of the underlying genetic complexity of these aspects of human physiology. They have generally been studied by multiple approaches, including clinical reports of patient phenotypes, diagnostic studies through which candidate genes are selected based on promising variants, bioinformatics to extract biomarkers of epigenome-wide dysregulation, and functional examination of the underlying mechanisms in cell and animal models. These multipronged approaches have led to significant advances in RDEO research.

However, RDEO research faces unique obstacles, as the variability of epigenetic regulation leads to technical challenges in both experimental design and statistical analysis. In addition, heterogeneity of both clinical features and chromatin patterns complicates interpretation of the results of such studies. Despite the difficulties in studying RDEOs, emerging technologies provide new opportunities in this field. In particular, the findings generated from RDEOs can provide valuable insights into the pathophysiology of common complex diseases. This review highlights examples of RDEOs that illustrate the current challenges in this field of research, and evaluates strategies that can be employed to overcome them.

## 2 Major epigenetic mechanisms associated with RDEOs

RDEOs are driven by genetic variants that lead to epigenetic dysregulation, often closely associated with the underlying chromatic template. Typically, these genetic variants have been identified in the coding regions of epigenetic regulators, altering their protein function and the downstream epigenetic patterns that they establish. While many genes contribute to epigenetic regulation in a broad sense, this review will focus on the three main chromatin-related mechanisms underlying RDEOs, i.e., disruption of DNA methylation (DNAm), histone modifications, and the activities of chromatin remodelers.

**FIGURE 1:**
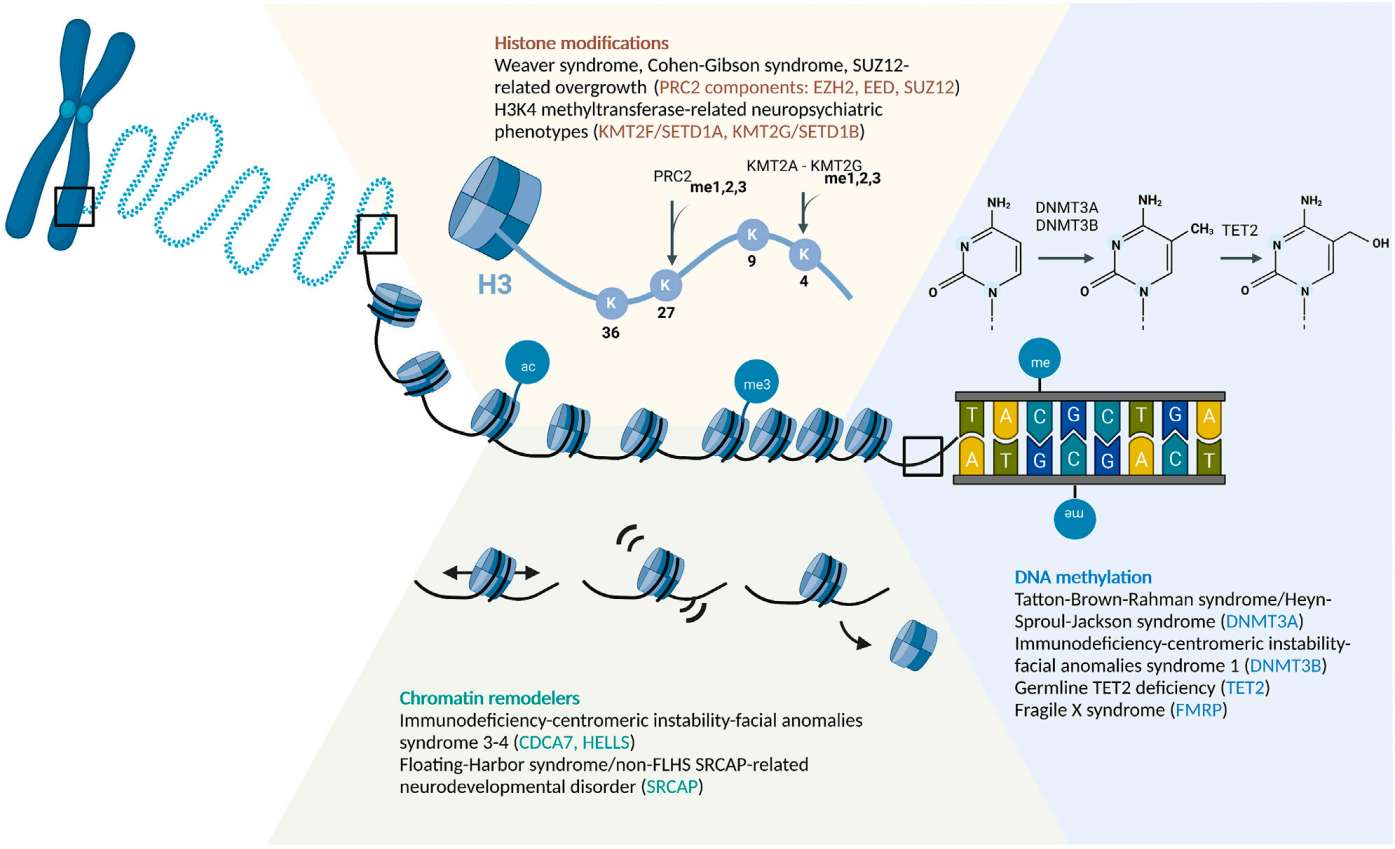
Schematic of Epigenetic Regulatory Mechanisms Associated With RDEOs: Chromatin Remodelers, Histone Modifications, and DNA Methylation. Abbreviations: ac, acetylation; me, methylation.

In the following sections, we will highlight some examples of RDEOs under each level of epigenetic control (Figure 1). This review does not represent a comprehensive overview of the RDEO literature, but discusses specific RDEOs chosen to highlight the challenges and novel opportunities in this field of research. A list of several known RDEOs registered in the OMIM database is included in Table 1 (Amberger et al., 2019).

**TABLE 1:** Known RDEOs and corresponding genetic origins and functional targets (if available). Curated based on information available in the OMIM database (https://www.omim.org).

Epigenetic regulation	Functional group	Gene	Target	RDEO	OMIM entry
DNA methylation	DNA methyltransferases (DMTs)	*DNMT1*	cytosine	Hereditary sensory neuropathy type IE (HSANIE)	# 614116
				Autosomal dominant cerebellar ataxia, deafness, and narcolepsy (ADCADN)	# 604121
		*DNMT3A*	cytosine	Tatton-Brown-Rahman syndrome (TBRS)	# 615879
				Heyn-Sproul-Jackson syndrome (HESJAS)	# 618724
		*DNMT3B*	cytosine	Immunodeficiency-centromeric instability-facial anomalies syndrome 1 (ICF1)	# 242860
	Methyl-CpG-binding proteins	*MBD5*	modified cytosine	Intellectual developmental disorder, autosomal dominant 1	# 156200
		*MECP2*	modified cytosine	Rett syndrome (RTT)	# 312750
				Rett syndrome-associated severe neonatal encephalopathy	# 300673
				X-linked syndromic intellectual developmental disorder-13 (MRXS13)	# 300055
				X-linked Lubs-type syndromic intellectual developmental disorder (MRXSL)	# 300260
	Methylcytosine dioxygenase (TETs)	*TET2*	modified cytosine	Immunodeficiency-75 (IMD75)	# 619126
		*TET3*	modified cytosine	Beck-Fahrner syndrome (BEFAHRS)	# 618798
	CGG repeats	*FMR1*		Fragile X syndrome (FXS)	# 300624
Histone modification	Lysine-specific methyltransferases (KMTs)	*KMT2A/MLL1*	H3K4	Wiedemann-Steiner syndrome (WDSTS)	# 605130
		*KMT2B/MLL2*	H3K4	Dystonia 28, childhood-onset (DYT28)	# 617284
		*KMT2C/MLL3*	H3K4	Kleefstra syndrome 2	# 617768
		*KMT2D/MLL4*	H3K4	Kabuki syndrome 1	
		*KMT2E/MLL5*	H3K4	O’Donnell-Luria-Rodan syndrome (ODLURO)	# 618512
		*KMT2F/SETD1A*	H3K4	Early-onset epilepsy with or without developmental delay (EPEDD)	# 618832
				Neurodevelopmental disorder with speech impairment and dysmorphic facies (NEDSID)	# 619056
		*KMT2G/SET1DB*	H3K4	Intellectual developmental disorder with seizures and language delay (IDDSELD)	# 611055
		*KMT2H/ASH1L*	H3K36	Autosomal dominant intellectual developmental disorder-52 (MRD52)	# 617796
		*KMT1D/EHMT1/GLP*	H3K9	Kleefstra syndrome 1 (KLEFS1)	# 610253
		*EED*	H3K27	Cohen-Gibson syndrome (COGIS)	# 617561
		*EZH2*	H3K27	Weaver syndrome (WVS)	# 277590
		*SUZ12*	H3K27	Imagawa-Matsumoto syndrome (IMMAS)/SUV12-related overgrowth	# 618786
		*KMT3A/SETD2*	H3K36	Luscan-Lumish syndrome (LLS)	# 616831
		*KMT3B/NSD1*	H3K36	Sotos syndrome (SOTOS)	# 117550
		*KMT3G/NSD2*	H3K36	Rauch-Steindl syndrome (RAUST)	# 619695
		*SETD5*	H3K36	Autosomal dominant intellectual developmental disorder-23 (MRD23)	# 615761
		*KMT5B/SUV420H1*	H4K20	Autosomal dominant intellectual developmental disorder-51 (MRD51)	# 617788
	Lysine-specific demethylases (KDMs)	*KDM1A/LSD1*	H3K4me1/2, H3K9me1/2	Cleft palate, psychomotor retardation, and distinctive facial features (CPRF)	# 616728
		*KDM3B/JHDM2b*	H3K9me	Diets-Jongmans syndrome	# 618846
		*KDM4B/JMJD2B*	H3K9/H3K36me2/3	Autosomal dominant intellectual developmental disorder-65 (MRD65)	# 619320
		*KDM5B/JARID1B*	H3K4me1/2/3	Autosomal recessive intellectual developmental disorder-65 (MRT65)	# 618109
		*KDM5C/JARID1C/SMCX*	H3K4me2/3	Claes-Jensen type of X-linked syndromic intellectual developmental disorder (MRXSCJ)	# 300534
		*KDM6A/UTX*	H3K27me2/3	Kabuki syndrome 2	# 300867
		*KDM6B/JMJD3*	H3K27me2/3	Neurodevelopmental disorder with coarse facies and mild distal skeletal abnormalities (NEDCFSA)	# 618505
		*KDM7B/PHF8*	H3K9	Siderius-type X-linked syndromic intellectual developmental disorder (MRXSSD)	# 300263
	Histone acetyltransferases (HATs)	*KAT3A/CREBBP*	H2A; H2B; H3	Rubinstein-Taybi syndrome (RSTS1)	# 180849
				Menke-Hennekam syndrome-1 (MKHK1)	# 618332
		*KAT3B/EP300*	H2A; H2B; H3	Rubinstein-Taybi syndrome 2 (RSTS2)	# 613684
				Menke-Hennekam syndrome-1 (MKHK2)	# 618333
		*KAT5/TIP60*	H4; H2A	Neurodevelopmental disorder with dysmorphic facies, sleep disturbance, and brain abnormalities (NEDFASB)	# 619103
		*KAT6A/MYST3/MOZ*	H3K9	Arboleda-Tham syndrome (ARTHS)	# 616268
		*KAT6B/MYST4/MORF*	H3K9	Genitopatellar syndrome (GTPTS)	# 606170
				SBBYS variant of Ohdo syndrome (SBBYSS)	# 603736
		*KANSL1*	H4K16	Koolen de Vreis syndrome (KDVS)	
		*KAT8/MYST1/HMOF*	H4K16	Li-Ghorgani-Weisz-Hubshman syndrome (LIGOWS)	# 618974
	Histone deacetylase (HDAC)	*HDAC4*		neurodevelopmental disorder with central hypotonia and dysmorphic facies (NEDCHF)	# 619797
		*HDAC6*		X-linked dominant chondrodysplasia	# 300863
		*HDAC8*		Cornelia de Lange syndrome-5 (CDLS5)	# 300882
	Histone kinases	*RPS6KA3/RSK2*	H3S10	Coffin-Lowry syndrome (CLS)	# 303600
				X-linked intellectual developmental disorder-19 (XLID19)	# 300844
	Ubiquitination	*UBE2A*	H2B	Intellectual developmental disorder, X-linked syndromic, Nascimento type (MRXSN)	# 300860
	Histone deubiquitinase	*BAP1*	H2AK119ub	Kury-Isidor syndrome (KURIS)	# 619762
		*ASXL1*	H2AK119ub	Bohring-Opitz syndrome	# 605039
Chromatin remodeling	SWI/SNF family	*ATRX*	H3.3	X-linked alpha-thalassemia/mental retardation syndrome (ATRX)	# 301040
				X-linked intellectual disability-hypotonic facies syndrome-1 (MRXFH1)	# 309580
		*HELLS*		Immunodeficiency-centromeric instability-facial anomalies syndromes 4 (ICF4)	# 616911
		*CDCA7*		Immunodeficiency-centromeric instability-facial anomalies syndromes 3 (ICF3)	# 616910
	Transcription factor of CDCA7	*ZBTB24*		Immunodeficiency-centromeric instability-facial anomalies syndromes 3 (ICF2)	# 614069
	SWI/SNF family - BAF complex and associated factors	*ARID1B/BAF250B*		Coffin-Siris syndrome-1 (CSS1)	# 135900
		*ARID1A/BAF250A*		Coffin-Siris syndrome-2 (CSS2)	# 614607
		*SMARCB1/BAF47*		Coffin-Siris syndrome-3 (CSS3)	# 614608
		*SMARCA4/BRG1*		Coffin-Siris syndrome-4 (CSS4)	# 614609
		*SMARCE1/BAF57*		Coffin-Siris syndrome-5 (CSS5)	# 616938
		*ARID2/BAF200*		Coffin-Siris syndrome-6 (CSS6)	# 617808
		*DPF2/BAF45D*		Coffin-Siris syndrome-7 (CSS7)	# 618027
		*SMARCC2/BAF170*		Coffin-Siris syndrome-8 (CSS8)	# 618362
		*SOX11*		Coffin-Siris syndrome-9 (CSS9)	# 615866
		*SMARCA2/BRM*		Nicolaides-Baraitser syndrome (NCBRS)	# 601358
				Blepharophimosis-impaired intellectual development syndrome (BIS)	# 619293
		*ADNP*		Helsmoortel-Van der Aa syndrome (HVDAS)	# 615873
	CHD family	*CHD1*		Pilarowski-Björnsson syndrome (PILBOS)	# 617682
		*CHD2*		Developmental and epileptic encephalopathy-94 (DEE94)	# 615369
		*CHD5*		Parenti-Mignot neurodevelopmental syndrome (PMNDS)	# 619873
		*CHD7*		CHARGE syndrome	# 214800
		*CHD8*		Intellectual developmental disorder with autism and macrocephaly (IDDAM)	# 615032
	CHD family - NuRD/Mi-2 complex and associated proteins	*CHD3*		Snijders Blok-Campeau syndrome (SNIBCPS)	# 618205
		*CHD4*		Sifrim-Hitz-Weiss syndrome (SIHIWES)	# 617159
		*PHF6*		Börjeson-Forssman-Lehmann syndrome (BFLS)	# 301900
	ISWI family	*BPTF/NURF301*		Neurodevelopmental disorder with dysmorphic facies and distal limb anomalies (NEDDFL)	# 617755
	Human INO80 complex	*SRCAP*	H2A.Z	Floating Harbor syndrome (FLHS)	# 136140
				Developmental delay, hypotonia, musculoskeletal defects, and behavioral abnormalities (DEHMBA)	# 619595
Other epigenetic regulation	Cohesin	*NIPBL*		Cornelia de Lange syndrome-1 (CDLS1)	# 122470
		*SMC1A*		Cornelia de Lange syndrome-2 (CDLS2)	# 300590
		*SMC3*		Cornelia de Lange syndrome-3 (CDLS3)	# 610759
		*RAD21*		Cornelia de Lange syndrome-4 (CDLS4)	# 614701
	Histone variants	*HIST1H1E*		Rahman syndrome (RMNS)	# 617537
	Histone chaperone	*FACT/SUPT16H*	H2A–H2B dimer; H2A.X; H3.1–H4 dimer; H3.2–H4 dimer	Neurodevelopmental disorder with dysmorphic facies and thin corpus callosum (NEDDFAC)	# 619480

### 2.1 RDEOs and DNA methylation

DNAm is the most common and widely-investigated DNA modification in the human epigenome. While there are several other DNA modifications, the role of DNAm in RDEOs has been elucidated in the greatest detail (Schübeler, 2015). In the human epigenome, DNAm is commonly found at the 5th carbon of cytosine, forming 5-methylcytosine (5 mC), which is most often, but not exclusively, maintained in the context of cytosine-phosphate-guanine (CpG) dinucleotides (Schübeler, 2015). In concert with histone and chromatin modifications, DNAm facilitates maintenance of the state of gene expression within cells (Cedar and Bergman, 2009; Allis and Jenuwein, 2016; Carter and Zhao, 2021). As a simple overview, DNA methyltransferases, such as *DNMT3A*, *DNMT3B*, and *DNMT3L*, establish the pattern of DNAm, which is then maintained by *DNMT1* and associated proteins (Schübeler, 2015; Lyko, 2018). The ten-eleven translocation (TET) protein family is responsible for the iterative oxidation of 5 mC–5-hydroxymethylcytosine (5hmC), 5-formylcytosine, and then 5-carboxylcytosine, which can eventually be removed by thymine DNA glycosylase-mediated base excision repair (Wu and Zhang, 2017). Genetic variants that alter the functions of these regulatory proteins can drive global disruption of DNAm level, leading to dysregulation of differentiation and developmental trajectories (Liao et al., 2015; Izzo et al., 2020).

#### 2.1.1 *DNMT3A*-associated RDEOs

*DNMT3A* is the most commonly mutated gene in patients with hematopoietic malignancies (Ley et al., 2010; Xie et al., 2014). In humans, germline variants of *DNMT3A* have been linked to changes in both neurological and physical development. For example, constitutional loss-of-function (LoF) variants of *DNMT3A* lead to decreased global DNAm and altered hematopoiesis in a condition referred to as Tatton-Brown-Rahman syndrome (*TBRS*; *OMIM* #615879) (Smith et al., 2021). *TBRS* is characterized by generalized overgrowth, intellectual disability and a range of neurodivergent phenotypes, distinctive facial features, and an altered hematopoietic landscape (Tatton-Brown et al., 2017; 2018). In contrast, gain-of-function (GoF) variants of *DNMT3A* were shown to cause Heyn-Sproul-Jackson syndrome (*HESJAS*; *OMIM* #618724), a very rare condition reported in only three patients to date, all of whom presented with microcephalic primordial dwarfism (Heyn et al., 2019). Functional characterization of the disease-associated variant showed altered *DNMT3A* function preventing binding to di- and trimethylated histone H3 lysine 36 (*H3K36me2/3*) and driving increased DNAm in regions marked by *H3K27me3* and *H3K4me3* (Heyn et al., 2019). The dichotomy of *TBRS* and *HESJAS*, driven by *DNMT3A* LoF and GoF, respectively, represents an intriguing example of RDEO-associated phenotypic heterogeneity, which will be explored further below.

#### 2.1.2 *TET2*-associated RDEOs

*TET* proteins mediate the active DNA demethylation process in mammals (Wu and Zhang, 2017). Similar to *DNMT3A*, *TET2* mutations have been linked to a wide range of hematopoietic malignancies (Delhommeau et al., 2009; Jiang, 2020). In addition to cancer, the role of *TET2* dysregulation in development of RDEOs has been explored. Germline *TET2* variants have been reported to be associated with a number of diseases with various levels of severity in humans. In one study, a patient with a heterozygous truncation variant of *TET2* presented with delayed developmental milestones in early childhood. Elderly germline carriers of the same allele also exhibited CD8^+^ T-cell exhaustion, skewing toward terminally differentiated effector memory cells re-expressing *CD45RA* (TEMRA) (Kaasinen et al., 2019). In another study, patients homozygous for LoF *TET2* variants showed severe immunodeficiency, lymphadenopathy, hepatosplenomegaly, developmental delay, autoimmunity, and B-cell or T-cell lymphoma (Stremenova Spegarova et al., 2020). Class-switch recombination defects were also detected in *TET2*-deficient B-cell (Stremenova Spegarova et al., 2020). Elevated levels of DNAm were observed along with decreased DNA hydroxymethylation in both heterozygous and homozygous *TET2* LoF cases (Kaasinen et al., 2019; Stremenova Spegarova et al., 2020). Furthermore, a rare variant burden analysis showed that rare heterozygous *TET2* variants were strongly enriched in individuals with neurodegenerative diseases (Cochran et al., 2020). These observations link heterozygous *TET2* variants to long-term epigenetic dysregulation, leading to altered immune profiles and neurodegeneration later in life. In contrast, homozygous LoF variants of *TET2* result in drastic alterations in early life that lead to extreme immunodeficiency. Taken together, these observations highlight the role of *TET2*, as well as the effects of the timing and dosage of its expression, in the regulation of hematopoiesis and neuronal specification.

#### 2.1.3 Fragile X syndrome (FXS)

Fragile X syndrome (FXS; OMIM #300624) is one of the best-studied RDEOs, and is relatively common with a prevalence of roughly 1/7000 among males and 1/11,000 among females (Coffee et al., 2009; Lévesque et al., 2009; Hunter et al., 2014). Distinct from RDEOs with mutations in the coding regions of genes, FXS is driven by expansion of a trinucleotide CGG repeat (>200 copies) in the promoter of the *FMR1* gene, which leads to excessive DNAm and transcriptional silencing of FMR1 (Sutcliffe et al., 1992). Patients with FXS typically present with delayed developmental milestones, intellectual disability, and a range of other neurodivergent phenotypes (Hagerman et al., 2017). FXS is also the most common single-gene driver of autism spectrum disorder (ASD), contributing to about 5% of cases (Fyke and Velinov, 2021). *FMR1* is located on the X chromosome and has an X-linked inheritance pattern, and the phenotype is usually more severe in males with FXS (Hagerman et al., 2017).

*FMR1* encodes FMRP, an RNA-binding protein that is essential for translational regulation in neuronal dendrites. Patients with FXS show the presence of dense but immature dendritic spines at excitatory synapses (Hagerman et al., 2017). Several mechanisms of action have been proposed to explain the physiology of FXS. As FMRP downregulates the activity of group 1 and group 5 metabotropic glutamate receptors (mGluR1 and mGluR5, respectively), FMRP deficiency is associated with increased glutaminergic signaling (Wang et al., 2010; Darnell et al., 2011). Activation of mGluR1 drives increased dendritic translation, AMPA receptor internalization, and long-term depression (LTD) *via* a series of signaling cascades, including phosphoinositide-3 kinase (PI3K) complex-mediated signaling. FMRP has been studied from a variety of perspectives to elucidate the underlying pathophysiology of FXS, and the results have highlighted the challenges in development of therapeutic strategies for RDEOs.

### 2.2 RDEOs and histone modifications

DNA nucleotides are wrapped around an octamer of histone proteins to form the basic unit of chromatin, the nucleosome (Allis and Jenuwein, 2016). Posttranslational modifications, such as acetylation, methylation, phosphorylation, SUMOylation, and ubiquitination, are often found on the N- or C-terminal tails of histones, thereby altering their charge and structure (Stillman, 2018). This can affect how histones interact with each other, DNA, and various other chromatin-binding proteins. For example, the negatively charged acetyl group neutralizes the electrostatic interaction between the lysine-rich histone tail and the negatively charged DNA backbone, which can lead to looser nucleosome packaging and facilitate increased accessibility of DNA to the transcriptional machinery (Shahbazian and Grunstein, 2007). Histone modifications are highly correlated with chromatin states (Ernst and Kellis, 2012), nucleosome spacing and positioning (Valouev et al., 2011), and DNAm patterns (Cedar and Bergman, 2009; Allis and Jenuwein, 2016; Velasco et al., 2021). Together, these epigenetic marks maintain transcriptional control throughout the cell cycle, and are responsive to environmental stimuli (Allis and Jenuwein, 2016).

#### 2.2.1 PRC2-related overgrowth syndromes

Polycomb repressive complex 2 (PRC2) is one of the best-studied epigenetic writers, and is responsible for *H3K27me1/2/3* (Margueron and Reinberg, 2011; Schuettengruber et al., 2017; Laugesen et al., 2019; van Mierlo et al., 2019). In particular, *H3K27me3* is considered the hallmark of PRC2-mediated repression (Laugesen et al., 2019). This mark is highly enriched in the promoters of silenced genes and poised enhancers (Rada-Iglesias et al., 2011). When present together with *H3K4me3*, a mark of active promoters, the chromatin is in a bivalent state that can readily switch between active and repressed transcriptional states, and the genes in the bivalent chromatin tend to be critical for early embryogenesis (Bernstein et al., 2006; Rada-Iglesias et al., 2011; Allis and Jenuwein, 2016; Liu et al., 2016; Hörmanseder et al., 2017). PRC2 maintains *H3K27* methylation status through cell division, and thereby plays a pivotal role in the establishment and preservation of cell identity during development (Rada-Iglesias et al., 2011; Schuettengruber et al., 2017). The PRC2 core complex is composed of four core subunits, i.e., EZH1/2, EED, SUZ12, and RBBP4/7 (van Mierlo et al., 2019), and non-conservative variants in these complexes have been shown to cause PRC2-related overgrowth syndromes: Weaver syndrome is linked to EZH2 variants (OMIM #277590; Gibson et al., 2012), Cohen–Gibson syndrome is related to EED variants (OMIM #617561; Cohen et al., 2015), and SUZ12-related overgrowth is linked to SUZ12 variants (OMIM #618786; Cyrus et al., 2019). Each of these PRC2-related overgrowth syndromes presents with tall stature, macrocephaly, advanced bone age, and intellectual disability, and Weaver syndrome and SUZ12-related overgrowth have been linked to increased risk of hematological malignancies and other cancers (Tatton-Brown et al., 2018; Cyrus et al., 2019; Gamu and Gibson, 2020). The importance of PRC2 in establishing transcriptional silencing in key developmental genes, such as the Hox gene clusters, can at least in part explain the correlation between LoF variants in these genes and overgrowth phenotypes (Gentile and Kmita, 2020). These phenomena highlight the non-redundancy of PRC2 subunits in facilitating histone modifications.

#### 2.2.2 *H3K4* methyltransferase-related neuropsychiatric phenotypes

*KMT2F* (also *SETD1A*) and *KMT2G* (also *SETD1B*) are SET1-family proteins, which are components of the Set1/COMPASS methyltransferase complexes that contribute to *H3K4me1/2/3* (Shilatifard, 2012). *H3K4me1* is highly enriched in enhancer regions, whereas *H3K4me3* is positively correlated with transcriptionally active promoters (Collins et al., 2019). H3K4 methylation has been shown to be vital for memory formation and retrieval (Gupta et al., 2010; Collins et al., 2019). Eight H3K4-specific histone lysine methyltransferases (KMTs) have been identified in humans to date (Allis et al., 2007), all of which are associated with neurological or psychiatric disorders (Collins et al., 2019). Hypomorphic *KMT2F* and *KMT2G* variants have been linked to neuropsychiatric disorders. Genome-wide screening and analysis of *de novo* insertion/deletion variant transmission pattern identified KMT2F as a candidate susceptibility gene for schizophrenia (Takata et al., 2014), which was supported by a meta-analysis with 1,077 parent–proband trios (Singh et al., 2016). In human neuronal cultures, a heterozygous LoF variant of *KMT2F* results in increased dendritic length and complexity, as well as increased neuronal bursting activity (Wang et al., 2022). The altered neuronal morphology and activity may underlie *KMT2F*-associated schizophrenia. In addition, both *KMT2F* and *KMT2G* variants are associated with early-onset epilepsy; in the case of KMT2G, patients also present with developmental delay, intellectual disability, and ASD-like behaviors (Hiraide et al., 2018; Yu X. et al., 2019; Den et al., 2019; Roston et al., 2021; Weng et al., 2022). Functional magnetic resonance imaging (fMRI) showed that instead of language-related cortical regions, the precentral gyrus is activated in the brains of patients when performing language tasks (Weng et al., 2022). The molecular basis of the altered neural connectivity remains to be elucidated.

### 2.3 RDEOs and chromatin remodeling

ATP-dependent chromatin remodeling complexes comprise another major class of epigenetic regulators. There are four main families of chromatin remodelers in eukaryotes: SWI/SNF, ISWI, CHD, and INO80 (Längst and Manelyte, 2015). Complexes in all families share the ability to bind to nucleosomes and break the DNA–histone interaction (Wang et al., 2007). Some remodeler families exhibit more specialized functions, such as the SWI/SNF family complexes that are responsible for nucleosome sliding and increasing DNA accessibility (Cenik and Shilatifard, 2021). Other remodeler families have dynamic functions, such as the INO80 family remodelers, which are involved in histone variant deposition, transcriptional activation, and DNA repair (Längst and Manelyte, 2015). Moreover, as chromatin remodelers alter nucleosome organization and chromatin assembly, their functions also do affect the access of *DNMT, TET*, and histone-modifying proteins, thus indirectly regulating DNAm and histone modifications (Allis and Jenuwein, 2016). For example, deposition of the histone variant H2A.Z by the chromatin remodeler SRCAP is negatively correlated with DNAm level across plants and animals (Zilberman et al., 2008; Conerly et al., 2010; Zemach et al., 2010). As the chromatin remodelers influence chromatin accessibility, they also play important roles in development, and pathogenic variants of the members of these families often lead to developmental disorders and malignancies (Boerkoel et al., 2002; Wang et al., 2007; Alfert et al., 2019; Cenik and Shilatifard, 2021).

#### 2.3.1 SRCAP-associated RDEOs

SNF2-related CREBBP activator protein (SRCAP; OMIM #611421) is the core catalytic component of the SRCAP chromatin remodeling complex, which is responsible for the deposition of H2A.Z–H2B dimers into nucleosomes (Wong et al., 2007; Feng et al., 2018). In addition to its chromatin remodeling capacity, SRCAP acts as a transcriptional regulator (Monroy et al., 2001) and promotes the DNA damage response (Dong et al., 2014). Recent evidence has shown that SRCAP is also critical for cell cycle progression by recruiting cytokinesis regulators to the midbody (Messina et al., 2021). Variants of SRCAP have been identified in patients presenting with neurodevelopmental disorders (NDDs), and their relations with Floating-Harbor syndrome (FLHS; OMIM #136140) are particularly well defined. FLHS is an extremely rare RDEO with about 100 cases reported to date, which is characterized by short stature, delayed bone age, distinctive craniofacial features, and delayed language development (Hood et al., 2012). FLHS is driven by truncation variants in exons 33 and 34 of SRCAP (“FLHS locus”), upstream of the region encoding the AT-hook DNA-binding motifs. In contrast, SRCAP variants upstream of exon 33 have been linked to non-FLHS SRCAP-related NDD, presenting with distinct DNAm profiles and an alternative set of phenotypes, including behavioral, psychiatric, and musculoskeletal problems as well as hypotonia (Rots et al., 2021). The DNAm changes may be driven by their inverse relations with H2A.Z deposition (Zilberman et al., 2008; Zemach et al., 2010). A recent study showed that transposable insertion variants in the SRCAP gene lead to particularly severe conditions characterized by failure to thrive, hypotonia, developmental delay, seizures, ASD, and mood disorders (Zhao et al., 2022). The range of clinical presentations of SRCAP-associated RDEOs demonstrates the phenotypic heterogeneity of these diseases.

#### 2.3.2 Immunodeficiency with centromeric instability and facial anomalies syndrome (ICF)

Immunodeficiency with centromeric instability and facial anomalies syndrome (ICF) exemplifies the heterogeneity of genetic causes and clinical presentation of RDEOs. ICF is characterized by chromosomal instability, global developmental delay, and humoral immune deficiency that results in recurrent and often fatal infections (Kamae et al., 2018; Helfricht et al., 2020). The molecular hallmarks of ICF include chromosomal deletions or duplications, heterochromatin decondensation, and centromeric breakage (Robertson and Wolffe, 2000). ICF has been linked to hypomorphic variants in four genes: *DNMT3B* (ICF1; OMIM #242860), *ZBTB24* (ICF2; OMIM #614069), *CDCA7* (ICF3; OMIM #616910), and *HELLS* (ICF4; OMIM #616911) (Xu et al., 1999, 199; Vukic and Daxinger, 2019). Whereas *DNMT3B* directly modifies DNA methylation status, *CDCA7* and *HELLS* are chromatin remodelers. Despite the common etiology of low DNAm at pericentromeric satellite 2 and 3 repeats (Velasco et al., 2018), ICF2, 3, and 4 are driven by defects in chromatin remodelers or their expression; *CDCA7* and *HELLS* form an ATP-dependent nucleosome remodeling complex that catalyzes nucleosome sliding (Jenness et al., 2018), while *ZBTB24* is a transcriptional regulator that binds directly to the CDCA7 promoter and activates its transcription (Wu et al., 2016; Jenness et al., 2018). Mechanistically, the functions of these three proteins are intertwined, thus explaining the similar clinical phenotypes associated with mutations in their genes.

ATP-dependent nucleosome remodeling complexes and their roles in maintaining nucleosome accessibility have been implicated in DNA double-strand break (DSB) repair (Groth et al., 2007; Harrod et al., 2020). One type of DSB repair, non-homologous end-joining (NHEJ), is crucial for B-cell maturation by mediating class-switch recombination (Woodbine et al., 2014). Hypo- or agammaglobulinemia in patients with ICF2–4 can be driven by the NHEJ defect (Blanco-Betancourt et al., 2004; Unoki et al., 2018; He et al., 2020). In contrast, however, *DNMT3B* has not been implicated in NHEJ. Again, these observations suggest that the mechanism underlying DNAm dysregulation in ICF2–4 may be different from than in ICF1 (Dunican et al., 2015). The dimorphism in regulatory processes between ICF1 and ICF2–4 despite the shared clinical phenotypes presents a challenge in the study of RDEOs, and is explored in more detail below.

## 3 Challenges and opportunities in studying RDEOs

### 3.1 Cell type and tissue specificity of epigenetic data can confound disease status and must be studied in a relevant model

The specificity of epigenetic signatures for each cell type and tissue poses challenges in studying RDEOs not encountered in genomic research, including cellular heterogeneity and difficulties in obtaining the tissue or cell type of interest. As epigenetic mechanisms play important roles in defining cell type and cell function (Carter and Zhao, 2021; Janssen and Lorincz, 2022), RDEOs that are driven by alternative epigenetic states can present atypical cell differentiation and maturation patterns as well as altered epigenetic states in each cell population (Blanco-Betancourt et al., 2004; Lindsley et al., 2016; Kamae et al., 2018; Stremenova Spegarova et al., 2020). These phenomena pose unique problems in analyzing bulk tissue epigenetic data. In heterogeneous tissues, the sources of epigenetic variability can be driven by changes in either cell type composition or the epigenetic regulation within a given cell type, or a combination thereof, and two main methods have been applied to resolve these issues. Many groups have constructed algorithms for computationally estimating cell type proportions in complex tissues. Specifically, novel methods have been developed to deconvolute cell type-specific effects of diseases (Zheng et al., 2018; Rahmani et al., 2019), providing new avenues for delineating the effects of RDEOs on cell type proportion versus cell type-specific effects. It should be noted that these methods have high computing power requirements as they involve complex modeling with interaction terms, and so are more appropriate in cohorts with larger sample sizes or for dimension-reduced data.

In addition to deconvolution, which may suffer from predictor errors or the lack of appropriate reference data sets, many studies have opted to analyze sorted cell samples or to use a single-cell approach. Cell sorting requires the isolation of relevant cell types using techniques such as fluorescence-activated cell sorting (FACS) (Hines et al., 2014; Gasparoni et al., 2018). Alternatively, examination of the patient’s epigenome at the single-cell level can easily distinguish whether the differential regulation is due to compositional changes, cell-specific dysregulation, or a mixture of both (Angermueller et al., 2016; Cheung et al., 2018; Hui et al., 2018; Karemaker and Vermeulen, 2018; Carter and Zhao, 2021). One potential caveat of FACS for purified cells or single-cell analysis is that the tissue dissociation and sorting procedures have been shown to alter transcriptomic profiles (van den Brink et al., 2017). These effects of the sorting process on epigenetic profiles warrant further investigation. Moreover, current single-cell epigenomic technologies result in data degradation and limit the interpretability of the data, so improvements in the experimental and analytic pipelines will be necessary for general application (Ahn et al., 2021; Carter and Zhao, 2021). Overall, single-cell approaches can be used to distinguish the sources of epigenetic variations, which is necessary to gain an understanding of the mechanisms underlying RDEOs.

Animal models, tissue culture, and postmortem tissues are often used to study RDEOs. Some of the most significant strides in RDEO research have been made in model organisms (Mizuguchi et al., 2004; Hagerman et al., 2017; Greenberg et al., 2019; Gamu and Gibson, 2020; Izzo et al., 2020; Huisman et al., 2021; Smith et al., 2021), but not all of this research can be readily translated to humans. For example, mGluR1 signaling blockers showed therapeutic potential for FXS in animal trials, but the effects failed to replicate in clinical trials in adolescents or adults with FXS (Berry-Kravis et al., 2016). These discrepancies may be due to differences in the trajectories of mouse and human brain development and FMRP function (Hagerman et al., 2017). A study examining FMRP binding partners in a human forebrain organoid model showed that, in addition to the presence of many human-specific FMRP binding partners, the organoid model could only be rescued by inhibition of PI3K and not mGluR1 (Kang et al., 2021). The human forebrain model utilized organoids, which are tiny progenitor cell-derived 3D structures in tissue culture that recapitulate basic tissue-level properties (Rossi et al., 2018). Compared to tissue cultures of immortalized cell lines, organoid technology promises to provide higher fidelity representation of complex tissue architecture and cell–cell interactions, with growth and regeneration properties more closely resembling those of primary tissues (Forsberg et al., 2018; Rossi et al., 2018). However, protocols for many organoid types have yet to be optimized; their formation can be unstable and they often fail to reach later stages of development (Rossi et al., 2018). Despite this caveat, organoid culture is an example of a method for mimicking human physiology, and represents an alternative approach for investigating RDEOs in biologically relevant cell models.

Similarly, patient-derived induced pluripotent stem cells (iPSCs) are also valuable for studying RDEOs with cell type specificity. A cocktail of transcription factors is used to convert somatic cells into pluripotent stem cells, which can then be further differentiated to address research questions with tissue specificity (Takahashi et al., 2007; Pozo et al., 2022; Sheridan et al., 2022). As iPSCs retain the donor genotype, they have been widely used to study RDs of genetic origin (Casanova et al., 2014). iPSCs in monolayer culture are more scalable and easier to maintain than 3D organoids, and so serve as better platforms for high-throughput genetic and drug screening (Mellios et al., 2018; Balafkan et al., 2020).

### 3.2 The developmental nature of many RDEOs leads to difficulty in timing of sample collection

In addition to cell specificity, epigenetic regulation is also sensitive to developmental timing. Most RDEOs have an early onset, as dysregulation of the epigenome affects the trajectory of cell fate decisions and cell identity in a critical period, which is often early embryogenesis (Butler et al., 2012; Lai et al., 2018; Calle-Fabregat et al., 2020; Izzo et al., 2020). Defects in these epigenetic regulatory mechanisms, therefore, lead to specific disease phenotypes that arise within a given developmental window. For example, *Dnmt3A* and *Dnmt3B* knockdown are lethal in mice at the embryonic stage, as their functions are critical for epigenetic reprogramming (Okano et al., 1999; Li, 2002). Similarly, null Kmt2d variants in mice are also lethal before embryonic day 10.5, as the protein encoded by this gene is essential for gastrulation (Ashokkumar et al., 2020). There is also evidence that the RDEO disease phenotype can be difficult to reverse past the early developmental period. This phenomenon has been demonstrated in clinical trials of candidate therapeutic agents for FXS, many of which have shown greater efficacy in children than in adolescents or adults (Berry-Kravis et al., 2016; Hagerman et al., 2017). Therefore, experimental conditions that resemble the relevant developmental time point may be crucial to extrapolate the findings for application to patient care.

Animal models are instrumental in understanding the effects of RDEOs on developmental trajectories beyond the molecular phenotypes. In contrast to cell-based models, model organisms can be used to examine morphological and behavioral anomalies as well as system-level dysregulation. In a mouse study, Vallianatos et al. (2020) showed that mutations in key regulators of *H3K4* methylation led to changes in dendritic morphology, memory formation, and behavioral aggression. This level of complexity speaks to the unique value of animal studies. Cell-based models, such as iPSCs, can also be used to mimic critical periods (Robinton and Daley, 2012). The process of creating iPSCs drives epigenetic reprogramming, thereby mimicking an embryonic state that can be examined as it is or after differentiation into specialized cell types (Scesa et al., 2021; Pozo et al., 2022). In addition, the use of primary patient samples, such as blood spots or amniotic fluid collected by amniocentesis, can facilitate the identification of RDEOs with biomarkers or be studied later to explore epigenetic dysregulation at an early stage.

Many clinical and experimental studies of RDEOs focus on early developmental time points, potentially due to the early onset of many of these diseases. For example, follow-up of patients into adulthood is comparatively rare, but such studies can be crucial to obtain a detailed clinical picture of RDEOs (Kodra et al., 2018). Studies of the natural history of RDEOs with complex clinical phenotypes can provide insights into the heterogeneity of symptoms and potential comorbidities, and thus inform long-term management and treatment solutions (Hagleitner et al., 2007; Arvio, 2016; Van Remmerden et al., 2020). Similar long-term observations can also be beneficial in animal studies. While animal models are often used to validate the clinical phenotypes observed in patients with RDEOs and validate the genotype–phenotype associations (Cacheiro et al., 2019), they can also be informative regarding potential disease progression. One example outside of RDEO is the report of B-cell malignancy in aging *Nfatc2* knockout (KO) mice before the discovery of the first human patient with homozygous LoF *NFATC2* variant, who developed B-cell lymphoma as a young adult (May et al., 2014; Sharma et al., 2022). Previous *Nfatc2* KO mouse studies mainly focused on skeletal and cartilage defects, as these symptoms manifest early (Ranger et al., 2000; Koga et al., 2005). Comprehensive analysis of RDEO animal models may also be instructive regarding the conditions of patients with corresponding variants. Qualitative studies of patients with RDEO and corresponding animal models can elucidate the effects of epigenetic dysregulation in sensitive tissues throughout the developmental trajectory.

### 3.3 RDEO analyses are inherently underpowered for high-dimensional omics analysis

Although there has been a great deal of progress in the discovery and characterization of RDEOs, technical limitations remain major challenges in the field. These challenges are exemplified in the epigenome-wide association study (EWAS), which is the most widely employed method of studying DNAm and uses multiple regression analysis to test for associations between measured DNAm sites and the phenotype of interest (Lappalainen and Greally, 2017; Li et al., 2019). An EWAS typically incorporates hundreds to thousands of samples, with measurement of hundreds of thousands of CpGs (Wahl et al., 2017; Merid et al., 2020). With multiple testing on all sites measured on the commonly employed Illumina EPIC BeadChip array ( ∼ 865 k), around 200 samples, with balanced cases and controls, would be needed to detect a 5% mean difference in methylation status at > 80% power, if a *p*-value threshold of 0.05 is set after correction for multiple comparisons (Mansell et al., 2019). In RDEOs, the case and control numbers are seldom balanced, leading to a higher type 1 error rate, or the identification of false-positive signals. In addition, the inherent rarity of RDEOs prevents recruitment of sufficient numbers of patients for fully powered analyses, except in the special case of differentially methylated CpGs that show strong and consistent disease-dependent effects, making type II error, or the identification of false-negatives, much more likely.

This problem has led to machine learning strategies and use of polyepigenetic predictors for analyzing RDEO DNAm data. For disease diagnosis, many groups focus on identifying and validating episignatures, i.e., polyepigenetic predictors that use a set of CpGs to estimate disease status (Aref-Eshghi et al., 2019; Sadikovic et al., 2019; Turinsky et al., 2020). To date, useful episignatures have been identified in 65 RDEOs (Levy et al., 2022). Similarly, the EpigenCentral web portal has been created to predict RDEO status using an algorithm that combines the classification results of three machine learning algorithms—penalized logistic regression, random forest, and support vector machine—for seven RDEOs as well as ASD and Down syndrome (Turinsky et al., 2020). However, the prediction accuracy for each disease requires additional validation. These and similar methods have the advantage that clustering-based analysis and machine learning-driven predictors avoid multiple testing, and instead focus on disease classification through the combination of smaller, relevant effects.

Other strategies to mitigate the problem of multiple testing include lessons learned from analysis of ChIP-Seq (chromatin immunoprecipitation followed by sequencing) and ATAC-Seq (assay for transposase-accessible chromatin followed by sequencing) data. Here, the problem of power can sometimes be overcome by summarizing test statistics and developing an analysis pipeline that typically involves chromatin state, motif, and enrichment analysis (Bardet et al., 2012; Steinhauser et al., 2016; Park et al., 2017). A similar strategy has been applied to DNAm data, where genomic regions with similar epigenetic regulation are grouped using R packages, such as CoMeBack and DMRcate (Peters et al., 2015; Gatev et al., 2020). These analyses summarize the high-dimensional data into limited values, so instead of hundreds of thousands of tests, only a few are performed.

There is a strong bias regarding which RDEOs are well-characterized and which are understudied (Ekins, 2017). The less common RDEOs are understudied not only due to funding constraints, but also because of the lack of access to samples. Animal models sometimes do not recapitulate the physiology of RDEOs (Berry-Kravis et al., 2016; Lui et al., 2016; Lui et al., 2018), and primary samples are difficult to obtain because of the rarity of these conditions. For example, both homozygous germline TET2 deficiency and HESJAS have been described in only one report each in the literature (Heyn et al., 2019; Stremenova Spegarova et al., 2020). Alternative bioinformatics approaches can be useful to identify dysregulated epigenetic elements in such cases. With the availability of publicly available data sets, and efforts to create reference epigenomes, such as the Encyclopedia of DNA Elements (ENCODE) project and the NIH Roadmap Epigenomics Project, it is becoming increasingly possible to create a reference epigenome for characterization of RDEOs (Kundaje et al., 2015). While the currently available reference epigenomes are diverse in the types of epigenetic marks examined and the tissues from which the data were generated, they nevertheless have small sample sizes and are low in demographic diversity. The International Human Epigenome Consortium (IHEC) web portal coordinates the creation and publication of reference epigenome maps from seven consortia, including ENCODE, Roadmap, and Blueprint (Bujold et al., 2016). At present, 178 whole-genome bisulfite sequencing (WGBS) data sets in primary human blood cells (including a variety of cell types) and 10 data sets in brain are available on IHEC. By comparing small disease cohorts against large healthy control groups, transcriptomics data have been used to pinpoint disease-associated genes in RDs with variants of unknown significance (Frésard et al., 2019; Ferraro et al., 2020). By expanding on the reference epigenome efforts and applying a similar outlier detection method to identify significantly dysregulated elements, future studies will better identify the downstream pathways that contribute to the pathophysiology of ultra-rare RDEOs.

### 3.4 Complex RDEO spectrum: Unknown gene–disease associations

Further complicating the technical challenges, it can be particularly difficult to elucidate the mechanisms driving RDEOs, as the interaction between genotype and phenotype is often unclear. With the rapid development of whole-genome sequencing technology, it has become increasingly feasible to determine the genetic variants present in patients with aberrant developmental trajectories. However, after sequencing, it can be difficult to determine the variants driving the disease or to delineate the link between the gene of interest and the phenotype in question.

For example, *DNMT3A* is necessary for *de novo* DNAm (Lyko, 2018). Differentiated cells derived from Dnmt3a-null mouse hematopoietic stem cells (HSCs) show a global loss of DNAm and deficient repression of HSC-specific genes, demonstrating its role in transcriptional silencing (Challen et al., 2012). These links between *DNMT3A* LoF and overgrowth phenotype, and between GoF variants and primordial dwarfism can be understood intuitively, but the relations between *DNMT3A* and brain subfunctions are less well understood. While some studies have demonstrated the necessity of *DNMT3A* expression for memory formation (LaPlant et al., 2010; Morris et al., 2013; Lavery et al., 2020) and emotional regulation (LaPlant et al., 2010; Elliott et al., 2016; Christian et al., 2020; Lavery et al., 2020), which were further supported by recent findings suggesting a role of non-CpG DNAm dysregulation as a mediator of these effects (Christian et al., 2020; Lavery et al., 2020), the regulatory mechanism has yet to be elucidated. This is mainly because *DNMT3A* regulates a wide variety of molecular pathways, and changes in its function can sometimes have a cascade effect as *DNMT3A* targets transcription factors, kinases, or other proteins that in turn exert regulatory functions. Although it is clear that several hundred genes are differentially expressed and methylated in the brains of *DNMT3A* LoF model mice (Christian et al., 2020; Lavery et al., 2020), the mechanisms underlying the functions of *DNMT3A* remain to be determined.

To elucidate the mechanisms underlying the regulatory disruptions in patients with RDEOs, it is crucial to identify downstream targets in relevant models and tissues. Targeted epigenomic or transcriptomic profiling in such models can be useful for identifying downstream dysregulated elements. For example, CLIP-Seq (crosslinking immunoprecipitation followed by sequencing) has been used to identify the RNA binding partners of FMRP to study FXS, as FMRP is an mRNA-binding protein. One recent study differentiated four brain cell types consisting of human dorsal and ventral forebrain neural progenitors and neurons, and applied integrated CLIP-Seq and transcriptomic analysis to determine FMRP targets (Li et al., 2020). The results showed that the neurogenesis pathway was consistently disrupted in all four cell types, while cell type-specific differential regulation of genes, such as *PIK3CB* and *SEC24C*, was identified and validated in dorsal neurons (Li et al., 2020). Interestingly, upregulation of the catalytic subunit of *PI3K, p110b*, has been reported to drive deficits in dendritic maturation and cognition in fmr1 knockout mice, and inhibition of p110b reversed the phenotypes associated with FXS (Gross et al., 2010; 2015). These findings were further validated in FXS forebrain organoids, and the results also identified PI3K as the main treatment target (Kang et al., 2021). Taken together, the extensive profiling of FMRP binding partners can generate a list of molecular targets for analysis of their therapeutic potential. This approach can be applied to studying other RDEOs, thus delineating the cascade effects of epigenetic dysregulation and identifying potential therapeutic targets.

### 3.5 Phenotypic heterogeneity indicates that a range of molecular pathways are affected in RDEOs

The phenotypic and genetic heterogeneity of RDEOs present further challenges in the study of these diseases. In most RDEOs, different patients with genetic variants in the same gene will present a spectrum of clinical phenotypes. Conversely, variants in functionally distinct genes can either lead to the same RDEO or drive similar clinical presentations. While these phenomena complicate the analysis and interpretation of experimental results, a number of tools are available to overcome these challenges. One of the simplest examples illustrating the heterogeneity of RDEO is the apparent reciprocity of the phenotypes associated with GoF versus LoF variants. For example, TBRS and HESJA are driven by LoF and GoF variants of DNMT3A, respectively (Heyn et al., 2019; Smith et al., 2021). The effect of a given variant can be established by a series of functional studies to explore the expression levels of the gene product and downstream target. For example, a global increase in DNAm was demonstrated in germline TET2 LoF disorder, which is consistent with the established function of TET2 in the DNA demethylation pathway (Wu and Zhang, 2017; Stremenova Spegarova et al., 2020).

In addition to the direct contrast of phenotypes attributable to GoF versus LoF variants, the genetic variants can sometimes drive differing phenotypes depending on their location within specific protein domains. For example, variants in two paralogous genes—CREBBP and EP300—are linked to two separate RDEOs; whereas variants outside of exon 30 and 31 of either gene are linked to Rubenstein-Taybi syndrome (RSTS), those within the two exons cause Menke-Hennekam syndrome (MKHK) with distinct clinical phenotypes and DNAm signatures (Bedford et al., 2010; Menke et al., 2016; 2018; Levy et al., 2022). Depending on the functions of the affected domain, variants in the same gene can drive significant phenotypic heterogeneity. Similarly, FLHS is driven by truncation variants in exons 33 and 34 of SRCAP, known as the FLHS locus (Hood et al., 2012; Nikkel et al., 2013; Seifert et al., 2014), whereas non-FLHS SRCAP-related NDD is linked to truncation variants proximal to the FLHS locus (Rots et al., 2021). Although all of the abovementioned SRCAP variants are likely to be non-functional, patients can present with a spectrum of clinical phenotypes, and again functional studies in relevant tissue contexts are warranted to further explore the underlying causes of this phenotypic heterogeneity. The functional differences of the SRCAP variants can drive different epigenome-wide profiles depending on the affected domain. To characterize FLHS SRCAP variants, Greenberg et al. assessed not only the effects of the variants on craniofacial development, but also evaluated the deposition of H2A.Z (a direct downstream target of SRCAP), by ChIP-Seq analyses. FLHS-associated variants were shown to disrupt the nuclear localization of SRCAP and prevent the deposition of H2A.Z.2, a subtype of H2A.Z, demonstrating that the variant is associated with SRCAP LoF (Greenberg et al., 2019). As SRCAP is also a transcriptional activator, RNA-Seq was performed to examine transcriptomic disruption. The application of a similar experimental pipeline to other SRCAP variants may help to resolve the issue of SRCAP-related phenotypic heterogeneity.

Phenotypic heterogeneity can also present as differences in disease onset. While most of the RDEOs discussed above manifest early in life, germline variants of the RDEO-associated genes may also confer a risk of later-onset neurodegenerative diseases. For example, fragile X-associated tremor/ataxia syndrome (FXTAS) is a neurodegenerative disease that affects premutation carriers (55–200 CGG repeats) in the FMR1 gene (Hagerman and Hagerman, 2016; Cabal-Herrera et al., 2020). The symptoms of FXTAS include intention tremor, cerebellar gait ataxia, neuropathic pain, and memory or executive function deficits, which are primarily observed in men older than 50 years (Hagerman and Hagerman, 2016; Cabal-Herrera et al., 2020; Salcedo-Arellano et al., 2020), although heterozygous female premutation carriers may also be affected (Hunter et al., 1998). Interestingly, in contrast to the transcriptional silencing associated with full mutation expansion in FXS, FMR1 mRNA is transcriptionally upregulated by 2–8-fold in patients with FXTAS in comparison to healthy controls (Tassone et al., 2000; 2007). However, this upregulation is accompanied by translational defects, resulting in a decreased FMRP protein level, which is negatively correlated with the number of CGG repeats (Kenneson, 2001; Iliff et al., 2013; Schneider et al., 2020). The sex-specific presentation of the disease indicates that the decreased FMRP dosage contributes to the disease phenotype (Cabal-Herrera et al., 2020). In addition, while the FXTAS variants do not functionally impair early development, the delayed onset also suggests that they cause an accumulation of molecular defects in alternative pathways resulting in neurodegeneration later in life.

Other RDEO-associated genes have also been implicated in neurodegenerative disorders. A recent study showed that rare LoF or non-coding TET2 variants were significantly enriched in populations with early-onset Alzheimer’s disease (EOAD) and frontotemporal dementia (FTD) (Cochran et al., 2020). However, Tet2 loss has also been shown to be neuroprotective in a mouse model of Parkinson’s disease (PD) (Marshall et al., 2020). These conflicting results may be due to differences in experimental setup between studies, or the homeostasis of DNAm regulation may be crucial for brain health. Similarly, DNMT3B and SRCAP variants have also been linked to neurodegenerative disorders, including Alzheimer’s disease (AD), PD, and amyotrophic lateral sclerosis (ALS) (Chesi et al., 2013; de Bem et al., 2016; Chen et al., 2017; Pezzi et al., 2017; Vardarajan et al., 2017). Defects in the DNA damage response have been proposed as potential mechanisms underlying FXTAS (Hagerman and Hagerman, 2016), as DNAm damage and oxidative stress have been shown to induce cellular senescence and neurodegeneration (Coppedè and Migliore, 2015; Martínez-Cué and Rueda, 2020; Gonzalez-Hunt and Sanders, 2021). As FMRPs (Alpatov et al., 2014), TET2 (Feng et al., 2019), DNMT3B (Jin and Robertson, 2013; Shih et al., 2022), and SRCAP (Dong et al., 2014) are all involved in the DNA damage response, the associations between variants in these genes and neurodegenerative disorders may be mediated by altered DNA damaged responses, although further research is needed to establish this potentially shared pathway.

These observations highlight the functional importance of these genes and the harmful effects of associated epigenetic dysregulation. This dysregulation may manifest as epigenetic drift, a phenomenon where the epigenetic profile becomes increasingly variable with age as the epigenetic machinery fails to faithfully maintain regulation through mitosis (Jones et al., 2015; Hernando-Herraez et al., 2019; Bergstedt et al., 2022). It is plausible that variants in key epigenetic regulators exacerbate the rate at which this deterioration occurs. The variants can drive cellular changes that may not translate to clinical symptoms early in life but become apparent at later stages. For individuals with deleterious variants in these genes, biomarkers such as epigenetic clocks—bioinformatics predictors of the biological aging process (Horvath, 2013; Jones et al., 2015; Levine et al., 2018)—may be useful for monitoring disease progression. Acceleration of age-related epigenetic changes was shown to be associated with a diagnosis of ASD using an epigenetic clock trained specifically for children, thereby demonstrating the utility of the clock for detecting altered developmental trajectories in diseases (McEwen et al., 2020). Epigenetic clocks trained in adults can be used to detect aging trajectories later in life (Levine et al., 2018; Lu et al., 2019) and to monitor the processes of epigenetic dysregulation in individuals carrying damaging variants in key epigenetic regulators. This bioinformatics tool should be considered in future RDEO studies across the age spectrum.

### 3.6 Genetic heterogeneity of RDEOs May inform shared targets of distinct genes

Finally, genetic heterogeneity is also commonly observed in RDEOs. Prior to the availability of sequencing technologies to detect disease subtypes with unique genetic origins, diseases such as ICF disorders and PRC2-related overgrowth syndromes were classified as single entities due to the overlap in their clinical phenotypes. In the case of ICF, the functional links between genes driving different subtypes of RDEO are unclear. In ICF, the interactions among drivers of ICF2–4 have been well characterized, while their relations with DNMT3B, the driver of ICF1, remain unknown (Wu et al., 2016; Jenness et al., 2018). ChIP-Seq analyses showed that ICF1 and ICF2-associated variants colocalized to a similar set of genes, which are enriched in pathways involving cellular maintenance, DNA repair, and telomere function (Thompson et al., 2018). The results further suggest that ZBTB24 is necessary for loading of DNMT3B onto DNA (Thompson et al., 2018). While additional studies are required to further characterize these interactions, these observations showed that the identification of shared binding partners and dysregulated effects can be used to probe functional overlap in RDEOs with genetic heterogeneity.

In contrast, genes associated with PRC2-related overgrowth syndromes all encode components of PRC2 (van Mierlo et al., 2019), with defects in each component preventing the normal functioning of the complex. PRC2 subunits have highly coordinated functions: EZH1/2 binds to histone targets and acts as the main catalytic unit (Margueron et al., 2008; Jani et al., 2019); EED provides the epigenetic reader function, propagating transcriptional repression by binding to nucleosomes with H3K27me3 and H3K9me3 and enhancing EZH2 activity (Margueron et al., 2009; Antonysamy et al., 2013; Wu et al., 2013); SUZ12 both stabilizes and recruits PRC2 to chromatin (Choi et al., 2017; Højfeldt et al., 2018); RBBP4/7 are necessary for binding of PRC2 to genomic regions without preexisting H3K27 methylation (Schuettengruber et al., 2017; van Mierlo et al., 2019). Regardless of the distinct roles of each member of core PRC2, they are all essential for its function. Furthermore, PRC2-associated transcriptional regulation involves interactions that extend to other proteins or complexes, including PRC1 (Yu J.-R. et al., 2019), PR-DUB complex (Abdel-Wahab et al., 2012, 2; Balasubramani et al., 2015), and the PRC2 accessory proteins (Oksuz et al., 2018; Glancy et al., 2021). Variants in genes encoding or regulating these elements can also alter the functions of PRC2, and lead to clinical symptoms involving similar pathways to PRC2-related overgrowth syndromes (Gamu and Gibson, 2020). Given that epigenetic regulatory mechanisms are highly interconnected, from DNA and histone modifications to chromatin organization (Cedar and Bergman, 2009; Velasco et al., 2021; Janssen and Lorincz, 2022), it may be relevant to focus on the study of RDEO genes with shared functions. Algorithms can also contribute to the identification of RDEOs with shared clinical and molecular characteristics. GestaltMatcher, for example, uses a deep convolution neural network to classify RDs based on patient‛s facial phenotypes (Hsieh et al., 2022). Data from patients with the same syndromes but different underlying genetic causes were shown to cluster together; for example, subtypes of Kabuki syndrome did not form distinct clusters (Hsieh et al., 2022). Such algorithms can be used to identify RDEOs with overlapping molecular origins based on clinical features. With accumulation of findings regarding related genes and the application of network analyses, the commonality of downstream targets and key regulators should provide valuable insights into the molecular bases of RDEOs.

## 4 Extending the RDEO findings to common complex diseases

Rare monogenic diseases provide unique insights into the functions of the affected genes and the mechanisms underlying the disorders, and this knowledge can be applied to understanding common complex diseases. It can be difficult to study common diseases with complex etiologies, as disease risk is driven by a combination of genetic, environmental, and lifestyle factors. The existence of monogenic diseases, such as RDEOs, suggests that a key gene and its associated molecular pathways have crucial pathophysiological roles, thus focusing research on the gene and pathway with translation of relevant findings to common diseases. Schizophrenia, for example, is a common psychiatric disorder with an array of factors that contribute to its pathogenesis, ranging from genetics, prenatal complications, lifetime adversity, and substance use (McCutcheon et al., 2020). The monogenic nature of KMT2F-associated schizophrenia, however, informs the relevance of H3K4 methylation to the pathophysiology of schizophrenia. Furthermore, a genome-wide association study highlighted the link between genetic variants in the H3K4 methylation pathway and schizophrenia status, validating these molecular relations outside of the context of rare damaging variants (Nesbit et al., 2021). H3K4 methylation pattern is tightly linked to human glial cell differentiation (Shulha et al., 2013). Microglia are a type of glial cells that are responsible for immune defense and maintenance of the central nervous system (Ginhoux et al., 2013). They also regulate synaptic pruning, a process that is crucial for reorganization of the brain connectomes and healthy brain function (Sowell et al., 2003; Paolicelli et al., 2011; Sakai, 2020). Samples from schizophrenia patients show increased microglial activity, accompanied by aberrant synaptic pruning and lowered dendritic spine density (Glausier and Lewis, 2013; Sellgren et al., 2019). However, iPSC-derived neurons with KMT2F-associated schizophrenia variants showed increased dendritic length and complexity (Wang et al., 2022). The cellular phenotype resembles that of ASD (Hutsler and Zhang, 2010; Varghese et al., 2017; Weir et al., 2018), another common disorder that is often caused by altered H3K4 methylation (Shen et al., 2014; Collins et al., 2019). Despite conflicting findings, altered H3K4 methylation has consistently been shown to induce aberrant pruning activity and dendritic structure (Sellgren et al., 2019; Chen et al., 2022; Wang et al., 2022). Further exploration of the roles of H3K4 modifications in microglial function, dendritic pruning, brain network activity, and memory formation and retrieval will increase our understanding of not only KMT2F-associated schizophrenia, but also other forms of schizophrenia and ASD. Similarly, exploring the molecular basis of TET2-associated immunodeficiency and TBRS, both of which are strongly linked to increased risk of hematopoietic malignancy, will help to elucidate the roles of TET2 and DNMT3A mutations as drivers of cancer. These RDEOs can shed light on the function of a given gene in specific tissues throughout the developmental trajectory, thus providing a foundation for anchoring research regarding common complex diseases.

## 5 Future directions

**FIGURE 2:**
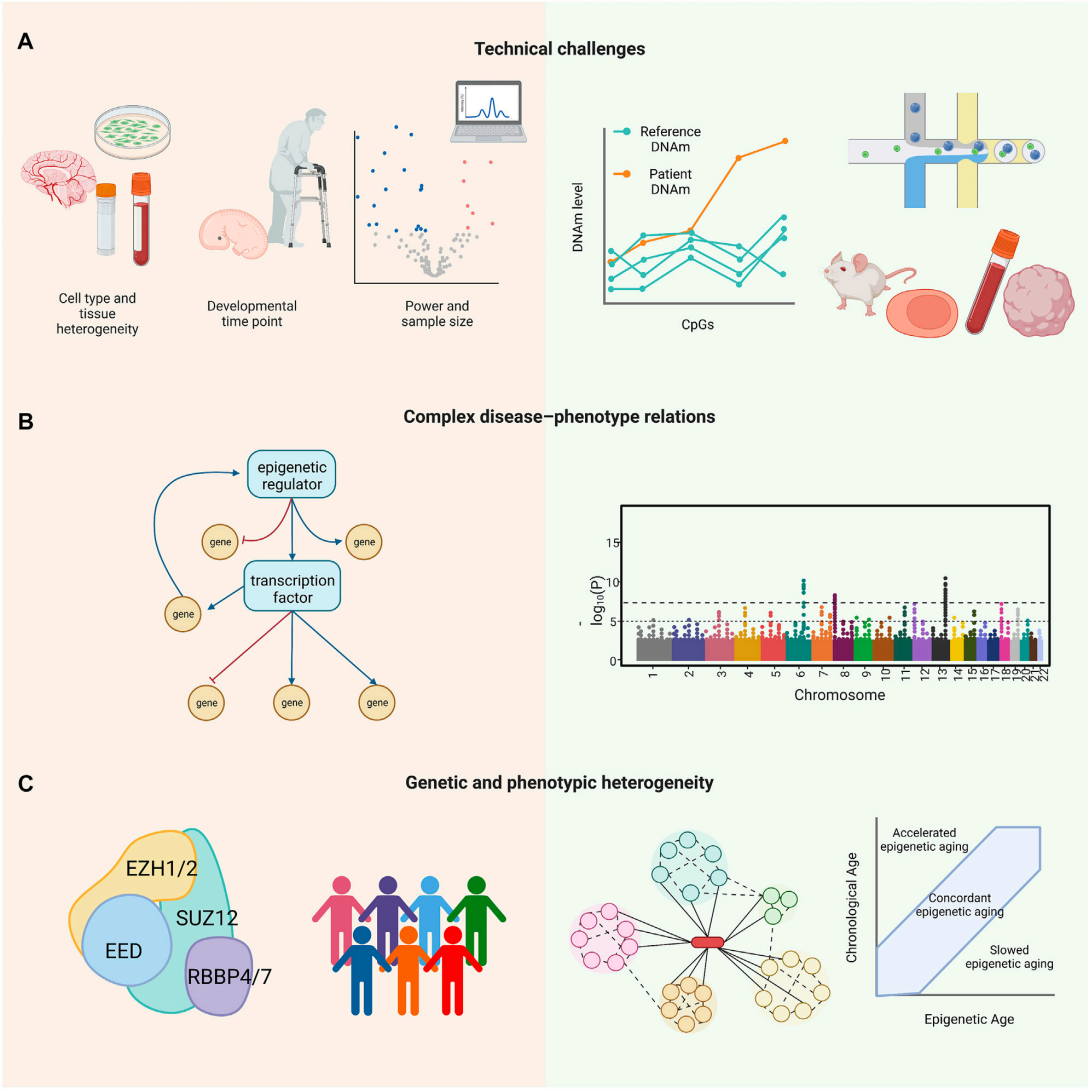
Challenges (left panel) and opportunities (right panel) in RDEO research. **(A)** Technical challenges include small sample size and low statistical power, cell type heterogeneity, and specificities of developmental time points. Example solutions include a reference epigenome, single-cell epigenomic profiling, and relevant RDEO models, such as animal models, primary samples, iPSCs, and organoids. **(B)** RDEOs often present with complex disease–phenotype relations, as the variants driving the RDEOs can drive widespread epigenetic dysregulation. Approaches such as RNA-Seq and EWAS can help to identify affected downstream elements and allow investigation of the mechanisms underlying the RDEO phenotype. **(C)** RDEOs commonly present with genetic and phenotypic heterogeneity. As an example of RDEO genetic heterogeneity, PRC2-related overgrowth syndromes are a group of RDEOs with a similar clinical phenotype all of which are linked to variants in PRC2. In contrast, patients with the same RDEO and variants in the same gene can have a range of clinical symptoms. Studies of interacting proteins in the same pathway can help to delineate the complexity of genetic heterogeneity, and tools such as the epigenetic clock may be applied to evaluate or monitor phenotypic heterogeneity. Abbreviations: CpGs, cytosine-guanine dinucleotides; DNAm, DNA methylation; RDEO, rare disease of epigenetic origin; EWAS, epigenome-wide association study; iPSC, induced pluripotent stem cell; PRC2, Polycomb repressive complex 2; RNA-Seq, RNA sequencing.

Several novel approaches have been developed to address the technical limitations of studying RDEOs and the complexities of these diseases (Figure 2). After identification of variants associated with RDEOs, basic research is required to characterize the molecules and mechanisms underlying the disease phenotypes. To address the problems of limited sample availability and cell type specificity, statistical tools and single-cell experiments in relevant tissues can be applied to facilitate detailed analysis. Early diagnosis using epigenome-wide profiles can facilitate the implementation of interventions through behavioral therapy, specialized learning programs, and individualized medicine. Epigenetic clocks may also be useful if applied to estimate cellular senescence and monitor disease progression in individuals with susceptible variants. Research on RDEOs can be further applied to understanding the roles of a single gene or factors that modify its activity in common complex diseases. The application of multidisciplinary research to RDEOs will facilitate the development of evidence-based treatments and management solutions. The development of tools such as the Matchmaker Exchange, DeepGestalt, and GestaltMatcher, which connect investigators studying the same genes or similar clinical phenotypes (Sobreira et al., 2015; Gurovich et al., 2019; Boycott et al., 2022; Hsieh et al., 2022), and efforts such as the Rare Diseases Models and Mechanism Network (Boycott et al., 2020) to connect basic and clinical researchers working on the same RDs will facilitate the investigation of RDEOs. In addition, health initiatives, such as national birth registries with postnatal dried blood spot collection or amniocentesis in individuals at higher risk, can facilitate early screening for RDEOs. This review highlighted the need to create a community of clinicians, patients, and researchers for the multidisciplinary study of RDEOs.

## Glossary

### Technologies for assaying molecular signals in rare disease research

ATAC-Seq (assay for transposase-accessible chromatin followed by sequencing): NGS technology used to identify accessible DNA regions by probing for open chromatin regions
ATAC-Seq is used to assess genome-wide chromatin accessibility in biological samples ChIP-Seq (chromatin immunoprecipitation followed by sequencing): NGS-based method to identify binding sites for DNA-associated proteins. The method involves crosslinking of DNA–protein complexes, precipitation of these complexes using an antibody against the protein of interest, and recovery of DNA fragments for sequencing
CLIP-Seq (crosslinking immunoprecipitation followed by sequencing): NGS-based method to identify binding sites of RNA-binding proteins. This method involves the *in vivo* crosslinking of RNA–protein complexes, precipitation of these complexes, and recovery of the RNA fragments for sequencing
RNA-Seq (RNA sequencing): NGS technology is used to detect and quantify mRNA molecules in biological samples
Single-cell sequencing: NGS method to assess sequencing information from individual cells. This involves the isolation of single cells before the recovery of DNA or RNA and amplification of the material for sequencing. Examples of single-cell sequencing technologies include single-cell RNA-Seq (scRNA-Seq), single-cell ATAC-Seq (scATAC-Seq), and single-cell CHIP-Seq (scCHIP-Seq)
DNAm microarray: High-throughput microarray for characterization of DNAm of bulk tissue after bisulfite conversion of unmodified cytosine residues to uracil. Converted DNA fragments are hybridized to probes on the array, followed by single-base extension of fluorescently tagged probes. The fluorescence pattern provides information of the level of DNAm at a given locus.

## References

[B1] Abdel-Wahab O. Adli M. LaFave L. M. Gao J. Hricik T. Shih A. H. (2012). ASXL1 mutations promote myeloid transformation through loss of PRC2-mediated gene repression. Cancer Cell 22, 180–193. 10.1016/j.ccr.2012.06.032 22897849 PMC3422511

[B2] Ahn J. Heo S. Lee J. Bang D. (2021). Introduction to single-cell DNA methylation profiling methods. Biomolecules 11, 1013. 10.3390/biom11071013 34356635 PMC8301785

[B3] Alfert A. Moreno N. Kerl K. (2019). The BAF complex in development and disease. Epigenetics Chromatin 12, 19. 10.1186/s13072-019-0264-y 30898143 PMC6427853

[B4] Allis C. D. Berger S. L. Cote J. Dent S. Jenuwien T. Kouzarides T. (2007). New nomenclature for chromatin-modifying enzymes. Cell 131, 633–636. 10.1016/j.cell.2007.10.039 18022353

[B5] Allis C. D. Jenuwein T. (2016). The molecular hallmarks of epigenetic control. Nat. Rev. Genet. 17, 487–500. 10.1038/nrg.2016.59 27346641

[B6] Alpatov R. Lesch B. J. Nakamoto-Kinoshita M. Blanco A. Chen S. Stützer A. (2014). A chromatin-dependent role of the Fragile X mental retardation protein FMRP in the DNA damage response. Cell 157, 869–881. 10.1016/j.cell.2014.03.040 24813610 PMC4038154

[B7] Amberger J. S. Bocchini C. A. Scott A. F. Hamosh A. (2019). OMIM.org: Leveraging knowledge across phenotype–gene relationships. Nucleic Acids Res. 47, D1038–D1043. 10.1093/nar/gky1151 30445645 PMC6323937

[B8] Angermueller C. Clark S. J. Lee H. J. Macaulay I. C. Teng M. J. Hu T. X. (2016). Parallel single-cell sequencing links transcriptional and epigenetic heterogeneity. Nat. Methods 13, 229–232. 10.1038/nmeth.3728 26752769 PMC4770512

[B9] Antonysamy S. Condon B. Druzina Z. Bonanno J. B. Gheyi T. Zhang F. (2013). Structural context of disease-associated mutations and putative mechanism of autoinhibition revealed by X-ray crystallographic analysis of the EZH2-SET domain. PLoS ONE 8, e84147. 10.1371/journal.pone.0084147 24367637 PMC3868555

[B10] Aref-Eshghi E. Bend E. G. Colaiacovo S. Caudle M. Chakrabarti R. Napier M. (2019). Diagnostic utility of genome-wide DNA methylation testing in genetically unsolved individuals with suspected hereditary conditions. Am. J. Hum. Genet. 104, 685–700. 10.1016/j.ajhg.2019.03.008 30929737 PMC6451739

[B11] Aristizabal M. J. Anreiter I. Halldorsdottir T. Odgers C. L. McDade T. W. Goldenberg A. (2020). Biological embedding of experience: A primer on epigenetics. Proc. Natl. Acad. Sci. U. S. A. 117, 23261–23269. 10.1073/pnas.1820838116 31624126 PMC7519272

[B12] Arvio M. (2016). Fragile-X syndrome – A 20-year follow-up study of male patients. Clin. Genet. 89, 55–59. 10.1111/cge.12639 26153079

[B13] Ashokkumar D. Zhang Q. Much C. Bledau A. S. Naumann R. Alexopoulou D. (2020). MLL4 is required after implantation whereas MLL3 becomes essential during late gestation. Development 147, 186999. 10.1242/dev.186999 32439762

[B14] Balafkan N. Mostafavi S. Schubert M. Siller R. Liang K. X. Sullivan G. (2020). A method for differentiating human induced pluripotent stem cells toward functional cardiomyocytes in 96-well microplates. Sci. Rep. 10, 18498. 10.1038/s41598-020-73656-2 33116175 PMC7595118

[B15] Balasubramani A. Larjo A. Bassein J. A. Chang X. Hastie R. B. Togher S. M. (2015). Cancer-associated ASXL1 mutations may act as gain-of-function mutations of the ASXL1–BAP1 complex. Nat. Commun. 6, 7307. 10.1038/ncomms8307 26095772 PMC4557297

[B16] Bardet A. F. He Q. Zeitlinger J. Stark A. (2012). A computational pipeline for comparative ChIP-seq analyses. Nat. Protoc. 7, 45–61. 10.1038/nprot.2011.420 22179591

[B17] Bedford D. C. Kasper L. H. Fukuyama T. Brindle P. K. (2010). Target gene context influences the transcriptional requirement for the KAT3 family of CBP and p300 histone acetyltransferases. Epigenetics 5, 9–15. 10.4161/epi.5.1.10449 20110770 PMC2829352

[B18] Bergstedt J. Azzou S. A. K. Tsuo K. Jaquaniello A. Urrutia A. Rotival M. (2022). The immune factors driving DNA methylation variation in human blood. Nat. Commun. 13, 5895. 10.1038/s41467-022-33511-6 36202838 PMC9537159

[B19] Bernstein B. E. Mikkelsen T. S. Xie X. Kamal M. Huebert D. J. Cuff J. (2006). A bivalent chromatin structure marks key developmental genes in embryonic stem cells. Cell 125, 315–326. 10.1016/j.cell.2006.02.041 16630819

[B20] Berry-Kravis E. Des Portes V. Hagerman R. Jacquemont S. Charles P. Visootsak J. (2016). Mavoglurant in fragile X syndrome: Results of two randomized, double-blind, placebo-controlled trials. Sci. Transl. Med. 8, 321ra5. 10.1126/scitranslmed.aab4109 26764156

[B21] Blanco-Betancourt C. E. Moncla A. Milili M. Jiang Y. L. Viegas-Péquignot E. M. Roquelaure B. (2004). Defective B-cell-negative selection and terminal differentiation in the ICF syndrome. Blood 103, 2683–2690. 10.1182/blood-2003-08-2632 14645008

[B22] Boerkoel C. F. Takashima H. John J. Yan J. Stankiewicz P. Rosenbarker L. (2002). Mutant chromatin remodeling protein SMARCAL1 causes Schimke immuno-osseous dysplasia. Nat. Genet. 30, 215–220. 10.1038/ng821 11799392

[B23] Boycott K. M. Azzariti D. R. Hamosh A. Rehm H. L. (2022). Seven years since the launch of the matchmaker exchange: The evolution of genomic matchmaking. Hum. Mutat. 43, 659–667. 10.1002/humu.24373 35537081 PMC9133175

[B24] Boycott K. M. Campeau P. M. Howley H. E. Pavlidis P. Rogic S. Oriel C. (2020). The Canadian rare diseases models and mechanisms (RDMM) network: Connecting understudied genes to model organisms. Am. J. Hum. Genet. 106, 143–152. 10.1016/j.ajhg.2020.01.009 32032513 PMC7010971

[B25] Boycott K. M. Hartley T. Biesecker L. G. Gibbs R. A. Innes A. M. Riess O. (2019). A diagnosis for all rare genetic diseases: The horizon and the next frontiers. Cell 177, 32–37. 10.1016/j.cell.2019.02.040 30901545

[B26] Bujold D. Morais D. A. de L. Gauthier C. Côté C. Caron M. Kwan T. (2016). The international human epigenome Consortium data portal. Cell Syst. 3, 496–499. 10.1016/j.cels.2016.10.019 27863956

[B27] Butler J. S. Koutelou E. Schibler A. C. Dent S. Y. (2012). Histone-modifying enzymes: Regulators of developmental decisions and drivers of human disease. Epigenomics 4, 163–177. 10.2217/epi.12.3 22449188 PMC3382990

[B28] Cabal-Herrera A. M. Tassanakijpanich N. Salcedo-Arellano M. J. Hagerman R. J. (2020). Fragile X-associated tremor/ataxia syndrome (FXTAS): Pathophysiology and clinical implications. Int. J. Mol. Sci. 21, 4391. 10.3390/ijms21124391 32575683 PMC7352421

[B29] Cacheiro P. Haendel M. A. Smedley D. Meehan T. Mason J. Mashhadi H. H. (2019). New models for human disease from the international mouse phenotyping Consortium. Mamm. Genome 30, 143–150. 10.1007/s00335-019-09804-5 31127358 PMC6606664

[B30] Calle-Fabregat C. de la Morante-Palacios O. Ballestar E. (2020). Understanding the relevance of DNA methylation changes in immune differentiation and disease. Genes 11, 110. 10.3390/genes11010110 31963661 PMC7017047

[B31] Carter B. Zhao K. (2021). The epigenetic basis of cellular heterogeneity. Nat. Rev. Genet. 22, 235–250. 10.1038/s41576-020-00300-0 33244170 PMC10880028

[B32] Casanova J.-L. Conley M. E. Seligman S. J. Abel L. Notarangelo L. D. (2014). Guidelines for genetic studies in single patients: Lessons from primary immunodeficiencies. J. Exp. Med. 211, 2137–2149. 10.1084/jem.20140520 25311508 PMC4203950

[B33] Cedar H. Bergman Y. (2009). Linking DNA methylation and histone modification: Patterns and paradigms. Nat. Rev. Genet. 10, 295–304. 10.1038/nrg2540 19308066

[B34] Cenik B. K. Shilatifard A. (2021). COMPASS and SWI/SNF complexes in development and disease. Nat. Rev. Genet. 22, 38–58. 10.1038/s41576-020-0278-0 32958894

[B35] Challen G. A. Sun D. Jeong M. Luo M. Jelinek J. Berg J. S. (2012). Dnmt3a is essential for hematopoietic stem cell differentiation. Nat. Genet. 44, 23–31. 10.1038/ng.1009 PMC3637952 22138693

[B36] Chen R. Liu Y. Djekidel M. N. Chen W. Bhattacherjee A. Chen Z. (2022). Cell type–specific mechanism of Setd1a heterozygosity in schizophrenia pathogenesis. Sci. Adv. 8, eabm1077. 10.1126/sciadv.abm1077 35245111 PMC8896793

[B37] Chen X. Xiao Y. Wei L. Wu Y. Lu J. Guo W. (2017). Association of DNMT3b gene variants with sporadic Parkinson’s disease in a Chinese Han population. J. Gene Med. 19, 360–365. 10.1002/jgm.2991 28990350

[B38] Chesi A. Staahl B. T. Jovičić A. Couthouis J. Fasolino M. Raphael A. R. (2013). Exome sequencing to identify de novo mutations in sporadic ALS trios. Nat. Neurosci. 16, 851–855. 10.1038/nn.3412 23708140 PMC3709464

[B39] Cheung P. Vallania F. Warsinske H. C. Donato M. Schaffert S. Chang S. E. (2018). Single-cell chromatin modification profiling reveals increased epigenetic variations with aging. Cell 173, 1385–1397. 10.1016/j.cell.2018.03.079 29706550 PMC5984186

[B40] Choi J. Bachmann A. L. Tauscher K. Benda C. Fierz B. Müller J. (2017). DNA binding by PHF1 prolongs PRC2 residence time on chromatin and thereby promotes H3K27 methylation. Nat. Struct. Mol. Biol. 24, 1039–1047. 10.1038/nsmb.3488 29058710

[B41] Christian D. L. Wu D. Y. Martin J. R. Moore J. R. Liu Y. R. Clemens A. W. (2020). DNMT3A haploinsufficiency results in behavioral deficits and global epigenomic dysregulation shared across neurodevelopmental disorders. Cell Rep. 33, 108416. 10.1016/j.celrep.2020.108416 33238114 PMC7716597

[B42] Cochran J. N. Geier E. G. Bonham L. W. Newberry J. S. Amaral M. D. Thompson M. L. (2020). Non-coding and loss-of-function coding variants in TET2 are associated with multiple neurodegenerative diseases. Am. J. Hum. Genet. 106, 632–645. 10.1016/j.ajhg.2020.03.010 32330418 PMC7212268

[B43] Coffee B. Keith K. Albizua I. Malone T. Mowrey J. Sherman S. L. (2009). Incidence of fragile X syndrome by newborn screening for methylated FMR1 DNA. Am. J. Hum. Genet. 85, 503–514. 10.1016/j.ajhg.2009.09.007 19804849 PMC2756550

[B44] Cohen A. S. A. Tuysuz B. Shen Y. Bhalla S. K. Jones S. J. M. Gibson W. T. (2015). A novel mutation in EED associated with overgrowth. J. Hum. Genet. 60, 339–342. 10.1038/jhg.2015.26 25787343

[B45] Collins B. E. Greer C. B. Coleman B. C. Sweatt J. D. (2019). Histone H3 lysine K4 methylation and its role in learning and memory. Epigenetics Chromatin 12, 7. 10.1186/s13072-018-0251-8 30616667 PMC6322263

[B46] Conerly M. L. Teves S. S. Diolaiti D. Ulrich M. Eisenman R. N. Henikoff S. (2010). Changes in H2A.Z occupancy and DNA methylation during B-cell lymphomagenesis. Genome Res. 20, 1383–1390. 10.1101/gr.106542.110 20709945 PMC2945187

[B47] Coppedè F. Migliore L. (2015). DNA damage in neurodegenerative diseases. Mutat. Res. 776, 84–97. 10.1016/j.mrfmmm.2014.11.010 26255941

[B48] Cyrus S. S. Cohen A. S. A. Agbahovbe R. Avela K. Yeung K. S. Chung B. H. Y. (2019). Rare SUZ12 variants commonly cause an overgrowth phenotype. Am. J. Med. Genet. 181, 532–547. 10.1002/ajmg.c.31748 31736240

[B49] Darnell J. C. Van Driesche S. J. Zhang C. Hung K. Y. S. Mele A. Fraser C. E. (2011). FMRP stalls ribosomal translocation on mRNAs linked to synaptic function and autism. Cell 146, 247–261. 10.1016/j.cell.2011.06.013 21784246 PMC3232425

[B50] de Bem C. M. B. E. Pezzi J. C. Borba E. M. Chaves M. L. F. de Andrade F. M. Fiegenbaum M. (2016). The synergistic risk effect of apolipoprotein ε4 and DNA (cytosine-5-)-methyltransferase 3 beta (DNMT3B) haplotype for Alzheimer’s disease. Mol. Biol. Rep. 43, 653–658. 10.1007/s11033-016-3999-6 27188425

[B51] Delhommeau F. Dupont S. Valle V. D. James C. Trannoy S. Massé A. (2009). Mutation in TET2 in myeloid cancers. N. Engl. J. Med. 360, 2289–2301. 10.1056/NEJMoa0810069 19474426

[B52] Den K. Kato M. Yamaguchi T. Miyatake S. Takata A. Mizuguchi T. (2019). A novel de novo frameshift variant in SETD1B causes epilepsy. J. Hum. Genet. 64, 821–827. 10.1038/s10038-019-0617-1 31110234

[B53] Dong S. Han J. Chen H. Liu T. Huen M. S. Y. Yang Y. (2014). The human SRCAP chromatin remodeling complex promotes DNA-end resection. Curr. Biol. 24, 2097–2110. 10.1016/j.cub.2014.07.081 25176633

[B54] Dunican D. S. Pennings S. Meehan R. R. (2015). Lsh is essential for maintaining global DNA methylation levels in amphibia and fish and interacts directly with Dnmt1. Biomed. Res. Int. 2015, 740637–740712. 10.1155/2015/740637 26491684 PMC4600896

[B55] Ekins S. (2017). Industrializing rare disease therapy discovery and development. Nat. Biotechnol. 35, 117–118. 10.1038/nbt.3787 28178258 PMC5320585

[B56] Elliott E. Manashirov S. Zwang R. Gil S. Tsoory M. Shemesh Y. (2016). Dnmt3a in the medial prefrontal cortex regulates anxiety-like behavior in adult mice. J. Neurosci. 36, 730–740. 10.1523/JNEUROSCI.0971-15.2016 26791204 PMC6601996

[B57] Ernst J. Kellis M. (2012). ChromHMM: Automating chromatin-state discovery and characterization. Nat. Methods 9, 215–216. 10.1038/nmeth.1906 22373907 PMC3577932

[B58] Feng S. Jacobsen S. E. Reik W. (2010). Epigenetic reprogramming in plant and animal development. Science 330, 622–627. 10.1126/science.1190614 21030646 PMC2989926

[B59] Feng Y. Li X. Cassady K. Zou Z. Zhang X. (2019). TET2 function in hematopoietic malignancies, immune regulation, and DNA repair. Front. Oncol. 9, 210. 10.3389/fonc.2019.00210 31001476 PMC6454012

[B60] Feng Y. Tian Y. Wu Z. Xu Y. (2018). Cryo-EM structure of human SRCAP complex. Cell Res. 28, 1121–1123. 10.1038/s41422-018-0102-y 30337683 PMC6218446

[B61] Ferraro N. M. Strober B. J. Einson J. Abell N. S. Aguet F. Barbeira A. N. (2020). Transcriptomic signatures across human tissues identify functional rare genetic variation. Science 369, eaaz5900. eaaz5900. 10.1126/science.aaz5900 32913073 PMC7646251

[B62] Forsberg S. L. Ilieva M. Maria Michel T. (2018). Epigenetics and cerebral organoids: Promising directions in autism spectrum disorders. Transl. Psychiatry 8, 14. 10.1038/s41398-017-0062-x 29317608 PMC5802583

[B63] Frésard L. Smail C. Ferraro N. M. Teran N. A. Li X. Smith K. S. (2019). Identification of rare-disease genes using blood transcriptome sequencing and large control cohorts. Nat. Med. 25, 911–919. 10.1038/s41591-019-0457-8 31160820 PMC6634302

[B64] Fyke W. Velinov M. (2021). FMR1 and autism, an intriguing connection revisited. Genes 12, 1218. 10.3390/genes12081218 34440392 PMC8394635

[B65] Gamu D. Gibson W. T. (2020). Reciprocal skeletal phenotypes of PRC2-related overgrowth and rubinstein–taybi syndromes: Potential role of H3K27 modifications. Cold Spring Harb. Mol. Case Stud. 6, a005058. 10.1101/mcs.a005058 32843427 PMC7476411

[B67] Gasparoni G. Bultmann S. Lutsik P. Kraus T. F. J. Sordon S. Vlcek J. (2018). DNA methylation analysis on purified neurons and glia dissects age and Alzheimer’s disease-specific changes in the human cortex. Epigenetics Chromatin 11, 41. 10.1186/s13072-018-0211-3 30045751 PMC6058387

[B68] Gatev E. Gladish N. Mostafavi S. Kobor M. S. (2020). CoMeBack: DNA methylation array data analysis for co-methylated regions. Bioinformatics 36, 2675–2683. 10.1093/bioinformatics/btaa049 31985744

[B69] Gentile C. Kmita M. (2020). Polycomb repressive complexes in Hox gene regulation: Silencing and beyond: The functional dynamics of polycomb repressive complexes in Hox gene regulation. BioEssays 42, 1900249. 10.1002/bies.201900249 32743818

[B70] Gibson W. T. Hood R. L. Zhan S. H. Bulman D. E. Fejes A. P. Moore R. (2012). Mutations in EZH2 cause Weaver syndrome. Am. J. Hum. Genet. 90, 110–118. 10.1016/j.ajhg.2011.11.018 22177091 PMC3257956

[B71] Ginhoux F. Lim S. Hoeffel G. Low D. Huber T. (2013). Origin and differentiation of microglia. Front. Cell. Neurosci. 7, 45. 10.3389/fncel.2013.00045 23616747 PMC3627983

[B72] Glancy E. Ciferri C. Bracken A. P. (2021). Structural basis for PRC2 engagement with chromatin. Curr. Opin. Struct. Biol. 67, 135–144. 10.1016/j.sbi.2020.10.017 33232890

[B73] Glausier J. R. Lewis D. A. (2013). Dendritic spine pathology in schizophrenia. Neuroscience 251, 90–107. 10.1016/j.neuroscience.2012.04.044 22546337 PMC3413758

[B74] Gonzalez-Hunt C. P. Sanders L. H. (2021). DNA damage and repair in Parkinson’s disease: Recent advances and new opportunities. J. Neurosci. Res. 99, 180–189. 10.1002/jnr.24592 32048327

[B75] Greenberg R. S. Long H. K. Swigut T. Wysocka J. (2019). Single amino acid change underlies distinct roles of H2A.Z subtypes in human syndrome. Cell 178, 1421–1436. 10.1016/j.cell.2019.08.002 31491386 PMC7103420

[B76] Gross C. Nakamoto M. Yao X. Chan C.-B. Yim S. Y. Ye K. (2010). Excess phosphoinositide 3-kinase subunit synthesis and activity as a novel therapeutic target in fragile X syndrome. J. Neurosci. 30, 10624–10638. 10.1523/JNEUROSCI.0402-10.2010 20702695 PMC2924772

[B77] Gross C. Raj N. Molinaro G. Allen A. G. Whyte A. J. Gibson J. R. (2015). Selective role of the catalytic PI3K subunit p110β in impaired higher order cognition in fragile X syndrome. Cell Rep. 11, 681–688. 10.1016/j.celrep.2015.03.065 25921527 PMC4426038

[B78] Groth A. Rocha W. Verreault A. Almouzni G. (2007). Chromatin challenges during DNA replication and repair. Cell 128, 721–733. 10.1016/j.cell.2007.01.030 17320509

[B79] Gupta S. Kim S. Y. Artis S. Molfese D. L. Schumacher A. Sweatt J. D. (2010). Histone methylation regulates memory formation. J. Neurosci. 30, 3589–3599. 10.1523/JNEUROSCI.3732-09.2010 20219993 PMC2859898

[B80] Gurovich Y. Hanani Y. Bar O. Nadav G. Fleischer N. Gelbman D. (2019). Identifying facial phenotypes of genetic disorders using deep learning. Nat. Med. 25, 60–64. 10.1038/s41591-018-0279-0 30617323

[B81] Hagerman R. J. Berry-Kravis E. Hazlett H. C. Bailey D. B. Moine H. Kooy R. F. (2017). Fragile X syndrome. Nat. Rev. Dis. Prim. 3, 17065. 10.1038/nrdp.2017.65 28960184

[B82] Hagerman R. J. Hagerman P. (2016). Fragile X-associated tremor/ataxia syndrome — Features, mechanisms and management. Nat. Rev. Neurol. 12, 403–412. 10.1038/nrneurol.2016.82 27340021

[B83] Hagleitner M. M. Lankester A. Maraschio P. Hulten M. Fryns J. P. Schuetz C. (2007). Clinical spectrum of immunodeficiency, centromeric instability and facial dysmorphism (ICF syndrome). J. Med. Genet. 45, 93–99. 10.1136/jmg.2007.053397 17893117

[B84] Harrod A. Lane K. A. Downs J. A. (2020). The role of the SWI/SNF chromatin remodelling complex in the response to DNA double strand breaks. DNA Repair 93, 102919. 10.1016/j.dnarep.2020.102919 33087260

[B85] He Y. Ren J. Xu X. Ni K. Schwader A. Finney R. (2020). Lsh/HELLS is required for B lymphocyte development and immunoglobulin class switch recombination. Proc. Natl. Acad. Sci. U. S. A. 117, 20100–20108. 10.1073/pnas.2004112117 32727902 PMC7443918

[B86] Helfricht A. Thijssen P. E. Rother M. B. Shah R. G. Du L. Takada S. (2020). Loss of ZBTB24 impairs nonhomologous end-joining and class-switch recombination in patients with ICF syndrome. J. Exp. Med. 217, e20191688. 10.1084/jem.20191688 32865561 PMC7526497

[B87] Henikoff S. Greally J. M. (2016). Epigenetics, cellular memory and gene regulation. Curr. Biol. 26, R644–R648. 10.1016/j.cub.2016.06.011 27458904

[B88] Hernando-Herraez I. Evano B. Stubbs T. Commere P.-H. Jan Bonder M. Clark S. (2019). Ageing affects DNA methylation drift and transcriptional cell-to-cell variability in mouse muscle stem cells. Nat. Commun. 10, 4361. 10.1038/s41467-019-12293-4 31554804 PMC6761124

[B89] Heyn P. Logan C. V. Fluteau A. Challis R. C. Auchynnikava T. Martin C.-A. (2019). Gain-of-function DNMT3A mutations cause microcephalic dwarfism and hypermethylation of Polycomb-regulated regions. Nat. Genet. 51, 96–105. 10.1038/s41588-018-0274-x 30478443 PMC6520989

[B90] Hines W. C. Su Y. Kuhn I. Polyak K. Bissell M. J. (2014). Sorting out the FACS: A devil in the details. Cell Rep. 6, 779–781. 10.1016/j.celrep.2014.02.021 24630040

[B91] Hiraide T. Nakashima M. Yamoto K. Fukuda T. Kato M. Ikeda H. (2018). De novo variants in SETD1B are associated with intellectual disability, epilepsy and autism. Hum. Genet. 137, 95–104. 10.1007/s00439-017-1863-y 29322246

[B92] Højfeldt J. W. Laugesen A. Willumsen B. M. Damhofer H. Hedehus L. Tvardovskiy A. (2018). Accurate H3K27 methylation can be established de novo by SUZ12-directed PRC2. Nat. Struct. Mol. Biol. 25, 225–232. 10.1038/s41594-018-0036-6 29483650 PMC5842896

[B93] Hood R. L. Lines M. A. Nikkel S. M. Schwartzentruber J. Beaulieu C. Nowaczyk M. J. M. (2012). Mutations in SRCAP, encoding SNF2-related CREBBP activator protein, cause floating-harbor syndrome. Am. J. Hum. Genet. 90, 308–313. 10.1016/j.ajhg.2011.12.001 22265015 PMC3276662

[B94] Hörmanseder E. Simeone A. Allen G. E. Bradshaw C. R. Figlmüller M. Gurdon J. (2017). H3K4 methylation-dependent memory of somatic cell identity inhibits reprogramming and development of nuclear transfer embryos. Cell Stem Cell 21, 135–143. 10.1016/j.stem.2017.03.003 28366589 PMC5505866

[B95] Horvath S. (2013). DNA methylation age of human tissues and cell types. Genome Biol. 14, R115. 10.1186/gb-2013-14-10-r115 24138928 PMC4015143

[B96] Hsieh T.-C. Bar-Haim A. Moosa S. Ehmke N. Gripp K. W. Pantel J. T. (2022). GestaltMatcher facilitates rare disease matching using facial phenotype descriptors. Nat. Genet. 54, 349–357. 10.1038/s41588-021-01010-x 35145301 PMC9272356

[B97] Hui T. Cao Q. Wegrzyn-Woltosz J. O’Neill K. Hammond C. A. Knapp D. J. H. F. (2018). High-resolution single-cell DNA methylation measurements reveal epigenetically distinct hematopoietic stem cell subpopulations. Stem Cell Rep. 11, 578–592. 10.1016/j.stemcr.2018.07.003 PMC6093082 30078558

[B98] Huisman C. Kim Y. A. Jeon S. Shin B. Choi J. Lim S. J. (2021). The histone H3-lysine 4-methyltransferase Mll4 regulates the development of growth hormone-releasing hormone-producing neurons in the mouse hypothalamus. Nat. Commun. 12, 256. 10.1038/s41467-020-20511-7 33431871 PMC7801453

[B99] Hunter J. Rivero-Arias O. Angelov A. Kim E. Fotheringham I. Leal J. (2014). Epidemiology of fragile X syndrome: A systematic review and meta-analysis. Am. J. Med. Genet. 164, 1648–1658. 10.1002/ajmg.a.36511 24700618

[B242] Hunter J. E. Berry-Kravis E. Hipp H. Todd P. K. (1998). “FMR1 Disorders,” in GeneReviews®. Editor Adam M. P. (Seattle, WA: University of Washington). Updated 2019 Nov 21. Available at: https://pubmed.ncbi.nlm.nih.gov/20301558/ . 20301558

[B100] Hutsler J. J. Zhang H. (2010). Increased dendritic spine densities on cortical projection neurons in autism spectrum disorders. Brain Res. 1309, 83–94. 10.1016/j.brainres.2009.09.120 19896929

[B101] Iliff A. J. Renoux A. J. Krans A. Usdin K. Sutton M. A. Todd P. K. (2013). Impaired activity-dependent FMRP translation and enhanced mGluR-dependent LTD in Fragile X premutation mice. Hum. Mol. Genet. 22, 1180–1192. 10.1093/hmg/dds525 23250915 PMC3578412

[B102] Izzo F. Lee S. C. Poran A. Chaligne R. Gaiti F. Gross B. (2020). DNA methylation disruption reshapes the hematopoietic differentiation landscape. Nat. Genet. 52, 378–387. 10.1038/s41588-020-0595-4 32203468 PMC7216752

[B103] Jani K. S. Jain S. U. Ge E. J. Diehl K. L. Lundgren S. M. Müller M. M. (2019). Histone H3 tail binds a unique sensing pocket in EZH2 to activate the PRC2 methyltransferase. Proc. Natl. Acad. Sci. U. S. A. 116, 8295–8300. 10.1073/pnas.1819029116 30967505 PMC6486736

[B104] Janssen S. M. Lorincz M. C. (2022). Interplay between chromatin marks in development and disease. Nat. Rev. Genet. 23, 137–153. 10.1038/s41576-021-00416-x 34608297

[B105] Jenness C. Giunta S. Müller M. M. Kimura H. Muir T. W. Funabiki H. (2018). HELLS and CDCA7 comprise a bipartite nucleosome remodeling complex defective in ICF syndrome. Proc. Natl. Acad. Sci. U. S. A. 115, E876. 10.1073/pnas.1717509115 29339483 PMC5798369

[B106] Jiang S. (2020). Tet2 at the interface between cancer and immunity. Commun. Biol. 3, 667. 10.1038/s42003-020-01391-5 33184433 PMC7661537

[B107] Jin B. Robertson K. D. (2013). “DNA methyltransferases, DNA damage repair, and cancer,” in Epigenetic alterations in oncogenesis advances in experimental medicine and biology. Editor Karpf A. R. (New York, NY: Springer), 3–29. 10.1007/978-1-4419-9967-2_1 PMC3707278 22956494

[B108] Jones M. J. Goodman S. J. Kobor M. S. (2015). DNA methylation and healthy human aging. Aging Cell 14, 924–932. 10.1111/acel.12349 25913071 PMC4693469

[B109] Kaasinen E. Kuismin O. Rajamäki K. Ristolainen H. Aavikko M. Kondelin J. (2019). Impact of constitutional TET2 haploinsufficiency on molecular and clinical phenotype in humans. Nat. Commun. 10, 1252. 10.1038/s41467-019-09198-7 30890702 PMC6424975

[B110] Kamae C. Imai K. Kato T. Okano T. Honma K. Nakagawa N. (2018). Clinical and immunological characterization of ICF syndrome in Japan. J. Clin. Immunol. 38, 927–937. 10.1007/s10875-018-0559-y 30353301

[B111] Kang Y. Zhou Y. Li Y. Han Y. Xu J. Niu W. (2021). A human forebrain organoid model of fragile X syndrome exhibits altered neurogenesis and highlights new treatment strategies. Nat. Neurosci. 24, 1377–1391. 10.1038/s41593-021-00913-6 34413513 PMC8484073

[B112] Karemaker I. D. Vermeulen M. (2018). Single-cell DNA methylation profiling: Technologies and biological applications. Trends Biotechnol. 36, 952–965. 10.1016/j.tibtech.2018.04.002 29724495

[B114] Kenneson A. Hagedorn C. H. Warren S. T. (2001). Reduced FMRP and increased FMR1 transcription is proportionally associated with CGG repeat number in intermediate-length and premutation carriers. Hum. Mol. Genet. 10, 1449–1454. 10.1093/hmg/10.14.1449 11448936

[B116] Kodra Y. Weinbach J. Posada-de-la-Paz M. Coi A. Lemonnier S. L. Van Enckevort D. (2018). Recommendations for improving the quality of rare disease registries. Int. J. Environ. Res. Public Health 15, 1644. 10.3390/ijerph15081644 30081484 PMC6121483

[B117] Koga T. Matsui Y. Asagiri M. Kodama T. de Crombrugghe B. Nakashima K. (2005). NFAT and Osterix cooperatively regulate bone formation. Nat. Med. 11, 880–885. 10.1038/nm1270 16041384

[B118] Kundaje A. Meuleman W. Ernst J. Bilenky M. Yen A. Heravi-Moussavi A. (2015). Integrative analysis of 111 reference human epigenomes. Nature 518, 317–330. 10.1038/nature14248 25693563 PMC4530010

[B119] Lai B. Gao W. Cui K. Xie W. Tang Q. Jin W. (2018). Principles of nucleosome organization revealed by single-cell micrococcal nuclease sequencing. Nature 562, 281–285. 10.1038/s41586-018-0567-3 30258225 PMC8353605

[B120] Längst G. Manelyte L. (2015). Chromatin remodelers: From function to dysfunction. Genes 6, 299–324. 10.3390/genes6020299 26075616 PMC4488666

[B121] LaPlant Q. Vialou V. Covington H. E. Dumitriu D. Feng J. Warren B. L. (2010). Dnmt3a regulates emotional behavior and spine plasticity in the nucleus accumbens. Nat. Neurosci. 13, 1137–1143. 10.1038/nn.2619 20729844 PMC2928863

[B122] Lappalainen T. Greally J. M. (2017). Associating cellular epigenetic models with human phenotypes. Nat. Rev. Genet. 18, 441–451. 10.1038/nrg.2017.32 28555657

[B123] Laugesen A. Højfeldt J. W. Helin K. (2019). Molecular mechanisms directing PRC2 recruitment and H3K27 methylation. Mol. Cell 74, 8–18. 10.1016/j.molcel.2019.03.011 30951652 PMC6452890

[B124] Lavery L. A. Ure K. Wan Y.-W. Luo C. Trostle A. J. Wang W. (2020). Losing Dnmt3a dependent methylation in inhibitory neurons impairs neural function by a mechanism impacting Rett syndrome. eLife 9, e52981. 10.7554/eLife.52981 32159514 PMC7065908

[B125] Lévesque S. Dombrowski C. Morel M.-L. Rehel R. Côté J.-S. Bussières J. (2009). Screening and instability of FMR1 alleles in a prospective sample of 24,449 mother–newborn pairs from the general population. Clin. Genet. 76, 511–523. 10.1111/j.1399-0004.2009.01237.x 19863547

[B126] Levine M. E. Lu A. T. Quach A. Chen B. H. Assimes T. L. Bandinelli S. (2018). An epigenetic biomarker of aging for lifespan and healthspan. Aging 10, 573–591. 10.18632/aging.101414 29676998 PMC5940111

[B127] Levy M. A. McConkey H. Kerkhof J. Barat-Houari M. Bargiacchi S. Biamino E. (2022). Novel diagnostic DNA methylation episignatures expand and refine the epigenetic landscapes of Mendelian disorders. HGG Adv. 3, 100075. 10.1016/j.xhgg.2021.100075 35047860 PMC8756545

[B128] Levy M. A. Relator R. McConkey H. Pranckeviciene E. Kerkhof J. Barat-Houari M. (2022). Functional correlation of genome-wide DNA methylation profiles in genetic neurodevelopmental disorders. Hum. Mutat. 43, 1609–1628. 10.1002/humu.24446 35904121

[B129] Ley T. J. Ding L. Walter M. J. McLellan M. D. Lamprecht T. Larson D. E. (2010). DNMT3A mutations in acute myeloid leukemia. N. Engl. J. Med. 363, 2424–2433. 10.1056/NEJMoa1005143 21067377 PMC3201818

[B130] Li E. (2002). Chromatin modification and epigenetic reprogramming in mammalian development. Nat. Rev. Genet. 3, 662–673. 10.1038/nrg887 12209141

[B131] Li M. Shin J. Risgaard R. D. Parries M. J. Wang J. Chasman D. (2020). Identification of FMR1-regulated molecular networks in human neurodevelopment. Genome Res. 30, 361–374. 10.1101/gr.251405.119 32179589 PMC7111522

[B132] Li M. Zou D. Li Z. Gao R. Sang J. Zhang Y. (2019). EWAS atlas: A curated knowledgebase of epigenome-wide association studies. Nucleic Acids Res. 47, D983–D988. 10.1093/nar/gky1027 30364969 PMC6324068

[B133] Liao J. Karnik R. Gu H. Ziller M. J. Clement K. Tsankov A. M. (2015). Targeted disruption of DNMT1, DNMT3A and DNMT3B in human embryonic stem cells. Nat. Genet. 47, 469–478. 10.1038/ng.3258 25822089 PMC4414868

[B134] Lindsley A. W. Saal H. M. Burrow T. A. Hopkin R. J. Shchelochkov O. Khandelwal P. (2016). Defects of B-cell terminal differentiation in patients with type-1 Kabuki syndrome. J. Allergy Clin. Immunol. 137, 179–187. 10.1016/j.jaci.2015.06.002 26194542 PMC4715656

[B135] Liu X. Wang C. Liu W. Li J. Li C. Kou X. (2016). Distinct features of H3K4me3 and H3K27me3 chromatin domains in pre-implantation embryos. Nature 537, 558–562. 10.1038/nature19362 27626379

[B136] Lu A. T. Quach A. Wilson J. G. Reiner A. P. Aviv A. Raj K. (2019). DNA methylation GrimAge strongly predicts lifespan and healthspan. Aging 11, 303–327. 10.18632/aging.101684 30669119 PMC6366976

[B137] Lui J. C. Barnes K. M. Dong L. Yue S. Graber E. Rapaport R. (2018). Ezh2 mutations found in the Weaver Overgrowth Syndrome cause a partial loss of H3K27 histone methyltransferase activity. J. Clin. Endocrinol. Metab. 103, 1470–1478. 10.1210/jc.2017-01948 29244146 PMC6276576

[B138] Lui J. C. Garrison P. Nguyen Q. Ad M. Keembiyehetty C. Chen W. (2016). EZH1 and EZH2 promote skeletal growth by repressing inhibitors of chondrocyte proliferation and hypertrophy. Nat. Commun. 7, 13685. 10.1038/ncomms13685 27897169 PMC5477487

[B139] Lyko F. (2018). The DNA methyltransferase family: A versatile toolkit for epigenetic regulation. Nat. Rev. Genet. 19, 81–92. 10.1038/nrg.2017.80 29033456

[B140] Mansell G. Gorrie-Stone T. J. Bao Y. Kumari M. Schalkwyk L. S. Mill J. (2019). Guidance for DNA methylation studies: Statistical insights from the Illumina EPIC array. BMC Genomics 20, 366. 10.1186/s12864-019-5761-7 31088362 PMC6518823

[B141] Margueron R. Justin N. Ohno K. Sharpe M. L. Son J. Drury W. J. III (2009). Role of the polycomb protein EED in the propagation of repressive histone marks. Nature 461, 762–767. 10.1038/nature08398 19767730 PMC3772642

[B142] Margueron R. Li G. Sarma K. Blais A. Zavadil J. Woodcock C. L. (2008). Ezh1 and Ezh2 maintain repressive chromatin through different mechanisms. Mol. Cell 32, 503–518. 10.1016/j.molcel.2008.11.004 19026781 PMC3641558

[B143] Margueron R. Reinberg D. (2011). The Polycomb complex PRC2 and its mark in life. Nature 469, 343–349. 10.1038/nature09784 21248841 PMC3760771

[B144] Marshall L. L. Killinger B. A. Ensink E. Li P. Li K. X. Cui W. (2020). Epigenomic analysis of Parkinson’s disease neurons identifies Tet2 loss as neuroprotective. Nat. Neurosci. 23, 1203–1214. 10.1038/s41593-020-0690-y 32807949

[B145] Martínez-Cué C. Rueda N. (2020). Cellular senescence in neurodegenerative diseases. Front. Cell. Neurosci. 14, 16. 10.3389/fncel.2020.00016 32116562 PMC7026683

[B146] May S. L. Zhou Q. Lewellen M. Carter C. M. Coffey D. Highfill S. L. (2014). Nfatc2 and Tob1 have non-overlapping function in T cell negative regulation and tumorigenesis. PLOS ONE 9, e100629. 10.1371/journal.pone.0100629 24945807 PMC4063948

[B147] McCutcheon R. A. Reis Marques T. Howes O. D. (2020). Schizophrenia—An overview. JAMA Psychiatry 77, 201–210. 10.1001/jamapsychiatry.2019.3360 31664453

[B148] McEwen L. M. O’Donnell K. J. McGill M. G. Edgar R. D. Jones M. J. MacIsaac J. L. (2020). The PedBE clock accurately estimates DNA methylation age in pediatric buccal cells. Proc. Natl. Acad. Sci. U. S. A. 117, 23329–23335. 10.1073/pnas.1820843116 31611402 PMC7519312

[B149] Mellios N. Feldman D. A. Sheridan S. D. Ip J. P. K. Kwok S. Amoah S. K. (2018). MeCP2-regulated miRNAs control early human neurogenesis through differential effects on ERK and AKT signaling. Mol. Psychiatry 23, 1051–1065. 10.1038/mp.2017.86 28439102 PMC5815944

[B150] Menke L. A. Study T. D. Gardeitchik T. Hammond P. Heimdal K. R. Houge G. (2018). Further delineation of an entity caused by CREBBP and EP300 mutations but not resembling Rubinstein–Taybi syndrome. Am. J. Med. Genet. A 176, 862–876. 10.1002/ajmg.a.38626 29460469

[B151] Menke L. A. van Belzen M. J. Alders M. Cristofoli F. Study T. D. Ehmke N. (2016). CREBBP mutations in individuals without Rubinstein–Taybi syndrome phenotype. Am. J. Med. Genet. A 170, 2681–2693. 10.1002/ajmg.a.37800 27311832

[B152] Merid S. K. Novoloaca A. Sharp G. C. Küpers L. K. Kho A. T. Roy R. (2020). Epigenome-wide meta-analysis of blood DNA methylation in newborns and children identifies numerous loci related to gestational age. Genome Med. 12, 25. 10.1186/s13073-020-0716-9 32114984 PMC7050134

[B154] Messina G. Prozzillo Y. Delle Monache F. Santopietro M. V. Atterrato M. T. Dimitri P. (2021). The ATPase SRCAP is associated with the mitotic apparatus, uncovering novel molecular aspects of Floating-Harbor syndrome. BMC Biol. 19, 184. 10.1186/s12915-021-01109-x 34474679 PMC8414691

[B155] Mizuguchi G. Shen X. Landry J. Wu W.-H. Sen S. Wu C. (2004). ATP-driven exchange of histone H2AZ variant catalyzed by SWR1 chromatin remodeling complex. Science 303, 343–348. 10.1126/science.1090701 14645854

[B156] Monroy M. A. Ruhl D. D. Xu X. Granner D. K. Yaciuk P. Chrivia J. C. (2001). Regulation of cAMP-responsive element-binding protein-mediated transcription by the SNF2/SWI-related protein, SRCAP. SRCAP. J. Biol. Chem. 276, 40721–40726. 10.1074/jbc.M103615200 11522779

[B157] Morris B. E. L. Henneberger R. Huber H. Moissl-Eichinger C. (2013). Microbial syntrophy: Interaction for the common good. FEMS Microbiol. Rev. 37, 384–406. 10.1111/1574-6976.12019 23480449

[B158] Nesbit N. Wallace R. Harihar S. Zhou M. Jung J.-Y. Silberstein M. (2021). Genomewide alteration of histone H3K4 methylation underlies genetic vulnerability to psychopathology. J. Genet. 100, 44. 10.1007/s12041-021-01294-2 34282735 PMC8459212

[B160] Nikkel S. M. Dauber A. de Munnik S. Connolly M. Hood R. L. Caluseriu O. (2013). The phenotype of floating-harbor syndrome: Clinical characterization of 52 individuals with mutations in exon 34 of SRCAP. Orphanet J. Rare Dis. 8, 63. 10.1186/1750-1172-8-63 23621943 PMC3659005

[B161] Okano M. Bell D. W. Haber D. A. Li E. (1999). DNA methyltransferases Dnmt3a and Dnmt3b are essential for de novo methylation and mammalian development. Cell 99, 247–257. 10.1016/S0092-8674(00)81656-6 10555141

[B162] Oksuz O. Narendra V. Lee C.-H. Descostes N. LeRoy G. Raviram R. (2018). Capturing the onset of PRC2-mediated repressive domain formation. Mol. Cell 70, 1149–1162. 10.1016/j.molcel.2018.05.023 29932905 PMC7700016

[B163] Paolicelli R. C. Bolasco G. Pagani F. Maggi L. Scianni M. Panzanelli P. (2011). Synaptic pruning by microglia is necessary for normal brain development. Science 333, 1456–1458. 10.1126/science.1202529 21778362

[B164] Park S.-J. Kim J.-H. Yoon B.-H. Kim S.-Y. (2017). A ChIP-Seq data analysis pipeline based on Bioconductor packages. Genomics Inf. 15, 11–18. 10.5808/GI.2017.15.1.11 PMC5389943 28416945

[B165] Peters T. J. Buckley M. J. Statham A. L. Pidsley R. Samaras K. V Lord R. (2015). De novo identification of differentially methylated regions in the human genome. Epigenetics Chromatin 8, 6. 10.1186/1756-8935-8-6 25972926 PMC4429355

[B166] Pezzi J. C. de Bem C. M. B. E. da Rocha T. J. Schumacher-Schuh A. F. Chaves M. L. F. Rieder C. R. (2017). Association between DNA methyltransferase gene polymorphism and Parkinson’s disease. Neurosci. Lett. 639, 146–150. 10.1016/j.neulet.2016.12.058 28041964

[B167] Pozo M. R. Meredith G. W. Entcheva E. (2022). Human iPSC-cardiomyocytes as an experimental model to study epigenetic modifiers of electrophysiology. Cells 11, 200. 10.3390/cells11020200 35053315 PMC8774228

[B168] Rada-Iglesias A. Bajpai R. Swigut T. Brugmann S. A. Flynn R. A. Wysocka J. (2011). A unique chromatin signature uncovers early developmental enhancers in humans. Nature 470, 279–283. 10.1038/nature09692 21160473 PMC4445674

[B169] Rahmani E. Schweiger R. Rhead B. Criswell L. A. Barcellos L. F. Eskin E. (2019). Cell-type-specific resolution epigenetics without the need for cell sorting or single-cell biology. Nat. Commun. 10, 3417. 10.1038/s41467-019-11052-9 31366909 PMC6668473

[B170] Ranger A. M. Gerstenfeld L. C. Wang J. Kon T. Bae H. Gravallese E. M. (2000). The nuclear factor of activated t cells (Nfat) transcription factor Nfatp (Nfatc2) is a repressor of chondrogenesis. J. Exp. Med. 191, 9–22. 10.1084/jem.191.1.9 10620601 PMC2195796

[B171] Richter T. Nestler-Parr S. Babela R. Khan Z. M. Tesoro T. Molsen E. (2015). Rare disease terminology and definitions—a systematic global review: Report of the ISPOR rare disease special interest group. Value Health 18, 906–914. 10.1016/j.jval.2015.05.008 26409619

[B172] Robertson K. D. Wolffe A. P. (2000). DNA methylation in health and disease. Nat. Rev. Genet. 1, 11–19. 10.1038/35049533 11262868

[B173] Robinton D. A. Daley G. Q. (2012). The promise of induced pluripotent stem cells in research and therapy. Nature 481, 295–305. 10.1038/nature10761 22258608 PMC3652331

[B174] Rodenhiser D. Mann M. (2006). Epigenetics and human disease: Translating basic biology into clinical applications. Can. Med. Assoc. J. 174, 341–348. 10.1503/cmaj.050774 16446478 PMC1373719

[B175] Rossi G. Manfrin A. Lutolf M. P. (2018). Progress and potential in organoid research. Nat. Rev. Genet. 19, 671–687. 10.1038/s41576-018-0051-9 30228295

[B176] Roston A. Evans D. Gill H. McKinnon M. Isidor B. Cogné B. (2021). SETD1B -associated neurodevelopmental disorder. J. Med. Genet. 58, 196–204. 10.1136/jmedgenet-2019-106756 32546566

[B177] Rots D. Chater-Diehl E. Dingemans A. J. M. Goodman S. J. Siu M. T. Cytrynbaum C. (2021). Truncating SRCAP variants outside the Floating-Harbor syndrome locus cause a distinct neurodevelopmental disorder with a specific DNA methylation signature. Am. J. Hum. Genet. 108, 1053–1068. 10.1016/j.ajhg.2021.04.008 33909990 PMC8206150

[B178] Sadikovic B. Aref-Eshghi E. Levy M. A. Rodenhiser D. (2019). DNA methylation signatures in mendelian developmental disorders as a diagnostic bridge between genotype and phenotype. Epigenomics 11, 563–575. 10.2217/epi-2018-0192 30875234

[B179] Sakai J. (2020). Core Concept: How synaptic pruning shapes neural wiring during development and, possibly, in disease. Proc. Natl. Acad. Sci. U. S. A. 117, 16096–16099. 10.1073/pnas.2010281117 32581125 PMC7368197

[B180] Salcedo-Arellano M. J. Dufour B. McLennan Y. Martinez-Cerdeno V. Hagerman R. (2020). Fragile X syndrome and associated disorders: Clinical aspects and pathology. Neurobiol. Dis. 136, 104740. 10.1016/j.nbd.2020.104740 31927143 PMC7027994

[B181] Scesa G. Adami R. Bottai D. (2021). iPSC preparation and epigenetic memory: Does the tissue origin matter? Cells 10, 1470. 10.3390/cells10061470 34208270 PMC8230744

[B182] Schneider A. Winarni T. I. Cabal-Herrera A. M. Bacalman S. Gane L. Hagerman P. (2020). Elevated FMR1-mRNA and lowered FMRP – a double-hit mechanism for psychiatric features in men with FMR1 premutations. Transl. Psychiatry 10, 205. 10.1038/s41398-020-00863-w 32576818 PMC7311546

[B183] Schübeler D. (2015). Function and information content of DNA methylation. Nature 517, 321–326. 10.1038/nature14192 25592537

[B184] Schuettengruber B. Bourbon H.-M. Di Croce L. Cavalli G. (2017). Genome regulation by polycomb and trithorax: 70 years and counting. Cell 171, 34–57. 10.1016/j.cell.2017.08.002 28938122

[B185] Seifert W. Meinecke P. Krüger G. Rossier E. Heinritz W. Wüsthof A. (2014). Expanded spectrum of exon 33 and 34 mutations in SRCAP and follow-up in patients with Floating-Harbor syndrome. BMC Med. Genet. 15, 127. 10.1186/s12881-014-0127-0 25433523 PMC4412025

[B186] Sellgren C. M. Gracias J. Watmuff B. Biag J. D. Thanos J. M. Whittredge P. B. (2019). Increased synapse elimination by microglia in schizophrenia patient-derived models of synaptic pruning. Nat. Neurosci. 22, 374–385. 10.1038/s41593-018-0334-7 30718903 PMC6410571

[B187] Shahbazian M. D. Grunstein M. (2007). Functions of site-specific histone acetylation and deacetylation. Annu. Rev. Biochem. 76, 75–100. 10.1146/annurev.biochem.76.052705.162114 17362198

[B188] Sharma M. Fu M. P. Lu H. Y. Sharma A. A. Modi B. P. Michalski C. (2022). Human complete NFAT1 deficiency causes a triad of joint contractures, osteochondromas, and B-cell malignancy. Blood 140, 1858–1874. 10.1182/blood.2022015674 35789258

[B189] Shen E. Shulha H. Weng Z. Akbarian S. (2014). Regulation of histone H3K4 methylation in brain development and disease. Phil. Trans. R. Soc. B 369, 20130514. 10.1098/rstb.2013.0514 25135975 PMC4142035

[B190] Sheridan S. D. Horng J. E. Perlis R. H. (2022). Patient-derived *in vitro* models of microglial function and synaptic engulfment in schizophrenia. Biol. Psychiatry 92, 470–479. 10.1016/j.biopsych.2022.01.004 35232567 PMC10039432

[B191] Shih H.-T. Chen W.-Y. Wang H.-Y. Chao T. Huang H.-D. Chou C.-H. (2022). DNMT3b protects centromere integrity by restricting R-loop-mediated DNA damage. Cell Death Dis. 13, 546. 10.1038/s41419-022-04989-1 35688824 PMC9187704

[B192] Shilatifard A. (2012). The COMPASS family of histone H3K4 methylases: Mechanisms of regulation in development and disease pathogenesis. Annu. Rev. Biochem. 81, 65–95. 10.1146/annurev-biochem-051710-134100 22663077 PMC4010150

[B193] Shulha H. P. Cheung I. Guo Y. Akbarian S. Weng Z. (2013). Coordinated cell type–specific epigenetic remodeling in prefrontal cortex begins before birth and continues into early adulthood. PLoS Genet. 9, e1003433. 10.1371/journal.pgen.1003433 23593028 PMC3623761

[B194] Singh T. Kurki M. I. Curtis D. Purcell S. M. Crooks L. McRae J. (2016). Rare loss-of-function variants in SETD1A are associated with schizophrenia and developmental disorders. Nat. Neurosci. 19, 571–577. 10.1038/nn.4267 26974950 PMC6689268

[B195] Smith A. M. LaValle T. A. Shinawi M. Ramakrishnan S. M. Abel H. J. Hill C. A. (2021). Functional and epigenetic phenotypes of humans and mice with DNMT3A Overgrowth Syndrome. Nat. Commun. 12, 4549. 10.1038/s41467-021-24800-7 34315901 PMC8316576

[B196] Sobreira N. Schiettecatte F. Valle D. Hamosh A. (2015). GeneMatcher: A matching tool for connecting investigators with an interest in the same gene. Hum. Mutat. 36, 928–930. 10.1002/humu.22844 26220891 PMC4833888

[B197] Sowell E. R. Peterson B. S. Thompson P. M. Welcome S. E. Henkenius A. L. Toga A. W. (2003). Mapping cortical change across the human life span. Nat. Neurosci. 6, 309–315. 10.1038/nn1008 12548289

[B198] Steinhauser S. Kurzawa N. Eils R. Herrmann C. (2016). A comprehensive comparison of tools for differential ChIP-seq analysis. Brief. Bioinforma. 17, 953–966. 10.1093/bib/bbv110 PMC5142015 26764273

[B199] Stillman B. (2018). Histone modifications: Insights into their influence on gene expression. Cell 175, 6–9. 10.1016/j.cell.2018.08.032 30217360

[B200] Stremenova Spegarova J. Lawless D. Mohamad S. M. B. Engelhardt K. R. Doody G. Shrimpton J. (2020). Germline TET2 loss of function causes childhood immunodeficiency and lymphoma. Blood 136, 1055–1066. 10.1182/blood.2020005844 32518946

[B201] Sutcliffe J. S. Nelson D. L. Zhang F. Pieretti M. Caskey C. T. Saxe D. (1992). DNA methylation represses FMR-1 transcription in fragile X syndrome. Hum. Mol. Genet. 1, 397–400. 10.1093/hmg/1.6.397 1301913

[B202] Takahashi K. Tanabe K. Ohnuki M. Narita M. Ichisaka T. Tomoda K. (2007). Induction of pluripotent stem cells from adult human fibroblasts by defined factors. Cell 131, 861–872. 10.1016/j.cell.2007.11.019 18035408

[B203] Takata A. Xu B. Ionita-Laza I. Roos J. L. Gogos J. A. Karayiorgou M. (2014). Loss-of-function variants in schizophrenia risk and SETD1A as a candidate susceptibility gene. Neuron 82, 773–780. 10.1016/j.neuron.2014.04.043 24853937 PMC4387883

[B205] Tassone F. Beilina A. Carosi C. Albertosi S. Bagni C. Li L. (2007). Elevated FMR1 mRNA in premutation carriers is due to increased transcription. RNA 13, 555–562. 10.1261/rna.280807 17283214 PMC1831862

[B206] Tassone F. Hagerman R. J. Taylor A. K. Gane L. W. Godfrey T. E. Hagerman P. J. (2000). Elevated levels of FMR1 mRNA in carrier males: A new mechanism of involvement in the fragile-X syndrome. Am. J. Hum. Genet. 66, 6–15. 10.1086/302720 10631132 PMC1288349

[B207] Tatton-Brown K. Loveday C. Yost S. Clarke M. Ramsay E. Zachariou A. (2017). Mutations in epigenetic regulation genes are a major cause of overgrowth with intellectual disability. Am. J. Hum. Genet. 100, 725–736. 10.1016/j.ajhg.2017.03.010 28475857 PMC5420355

[B208] Tatton-Brown K. Zachariou A. Loveday C. Renwick A. Mahamdallie S. Aksglaede L. (2018). The Tatton-Brown-rahman syndrome: A clinical study of 55 individuals with de novo constitutive DNMT3A variants. Wellcome Open Res. 3, 46. 10.12688/wellcomeopenres.14430.1 29900417 PMC5964628

[B209] Thompson J. J. Kaur R. Sosa C. P. Lee J.-H. Kashiwagi K. Zhou D. (2018). ZBTB24 is a transcriptional regulator that coordinates with DNMT3B to control DNA methylation. Nucleic Acids Res. 46, 10034–10051. 10.1093/nar/gky682 30085123 PMC6212772

[B210] Turinsky A. L. Choufani S. Lu K. Liu D. Mashouri P. Min D. (2020). EpigenCentral: Portal for DNA methylation data analysis and classification in rare diseases. Hum. Mutat. 41, 1722–1733. 10.1002/humu.24076 32623772

[B211] Unoki M. Funabiki H. Velasco G. Francastel C. Sasaki H. (2018). CDCA7 and HELLS mutations undermine nonhomologous end joining in centromeric instability syndrome. J. Clin. Invest. 129, 78–92. 10.1172/JCI99751 30307408 PMC6307953

[B212] Vallianatos C. N. Raines B. Porter R. S. Bonefas K. M. Wu M. C. Garay P. M. (2020). Mutually suppressive roles of KMT2A and KDM5C in behaviour, neuronal structure, and histone H3K4 methylation. Commun. Biol. 3, 278–314. 10.1038/s42003-020-1001-6 32483278 PMC7264178

[B213] Valouev A. Johnson S. M. Boyd S. D. Smith C. L. Fire A. Z. Sidow A. (2011). Determinants of nucleosome organization in primary human cells. Nature 474, 516–520. 10.1038/nature10002 21602827 PMC3212987

[B214] van den Brink S. C. Sage F. Vértesy Á. Spanjaard B. Peterson-Maduro J. Baron C. S. (2017). Single-cell sequencing reveals dissociation-induced gene expression in tissue subpopulations. Nat. Methods 14, 935–936. 10.1038/nmeth.4437 28960196

[B215] van Mierlo G. Veenstra G. J. C. Vermeulen M. Marks H. (2019). The complexity of PRC2 subcomplexes. Trends Cell Biol. 29, 660–671. 10.1016/j.tcb.2019.05.004 31178244

[B216] Van Remmerden M. C. Hoogland L. Mous S. E. Dierckx B. Coesmans M. Moll H. A. (2020). Growing up with fragile X syndrome: Concerns and care needs of young adult patients and their parents. J. Autism Dev. Disord. 50, 2174–2187. 10.1007/s10803-019-03973-7 30879259 PMC7261272

[B217] Vardarajan B. N. Tosto G. Lefort R. Yu L. Bennett D. A. De Jager P. L. (2017). Ultra-rare mutations in SRCAP segregate in Caribbean Hispanic families with Alzheimer disease. Neurol. Genet. 3, e178. 10.1212/NXG.0000000000000178 28852706 PMC5570674

[B218] Varghese M. Keshav N. Jacot-Descombes S. Warda T. Wicinski B. Dickstein D. L. (2017). Autism spectrum disorder: Neuropathology and animal models. Acta Neuropathol. 134, 537–566. 10.1007/s00401-017-1736-4 28584888 PMC5693718

[B219] Velasco G. Francastel C. (2019). Genetics meets DNA methylation in rare diseases. Clin. Genet. 95, 210–220. 10.1111/cge.13480 30456829

[B220] Velasco G. Grillo G. Touleimat N. Ferry L. Ivkovic I. Ribierre F. (2018). Comparative methylome analysis of ICF patients identifies heterochromatin loci that require ZBTB24, CDCA7 and HELLS for their methylated state. Hum. Mol. Genet. 27, 2409–2424. 10.1093/hmg/ddy130 29659838

[B221] Velasco G. Ulveling D. Rondeau S. Marzin P. Unoki M. Cormier-Daire V. (2021). Interplay between histone and DNA methylation seen through comparative methylomes in rare Mendelian disorders. Int. J. Mol. Sci. 22, 3735. 10.3390/ijms22073735 33916664 PMC8038329

[B222] Vukic M. Daxinger L. (2019). DNA methylation in disease: Immunodeficiency, Centromeric instability, Facial anomalies syndrome. Essays Biochem. 63, 773–783. 10.1042/EBC20190035 31724723 PMC6923317

[B223] Wahl S. Drong A. Lehne B. Loh M. Scott W. R. Kunze S. (2017). Epigenome-wide association study of body mass index, and the adverse outcomes of adiposity. Nature 541, 81–86. 10.1038/nature20784 28002404 PMC5570525

[B224] Wang G. G. Allis C. D. Chi P. (2007). Chromatin remodeling and cancer, part II: ATP-dependent chromatin remodeling. Trends Mol. Med. 13, 373–380. 10.1016/j.molmed.2007.07.004 17822959 PMC4337864

[B225] Wang L. W. Berry-Kravis E. Hagerman R. J. (2010). Fragile X: Leading the way for targeted treatments in autism. Neurotherapeutics 7, 264–274. 10.1016/j.nurt.2010.05.005 20643379 PMC4084556

[B226] Wang S. Rhijn J.-R. van Akkouh I. Kogo N. Maas N. Bleeck A. (2022). Loss-of-function variants in the schizophrenia risk gene SETD1A alter neuronal network activity in human neurons through the cAMP/PKA pathway. Cell Rep. 39, 110790. 10.1016/j.celrep.2022.110790 35508131 PMC7615788

[B227] Weir R. K. Bauman M. D. Jacobs B. Schumann C. M. (2018). Protracted dendritic growth in the typically developing human amygdala and increased spine density in young ASD brains. J. Comp. Neurol. 526, 262–274. 10.1002/cne.24332 28929566 PMC5728110

[B228] Weng R. Nenning K.-H. Schwarz M. Riedhammer K. M. Brunet T. Wagner M. (2022). Connectome analysis in an individual with SETD1B-related neurodevelopmental disorder and epilepsy. J. Dev. Behav. Pediatr. 43, e419–e422. 10.1097/DBP.0000000000001079 35385430

[B229] Wong M. M. Cox L. K. Chrivia J. C. (2007). The chromatin remodeling protein, SRCAP, is critical for deposition of the histone variant H2A.Z at promoters. J. Biol. Chem. 282, 26132–26139. 10.1074/jbc.M703418200 17617668

[B230] Woodbine L. Gennery A. R. Jeggo P. A. (2014). The clinical impact of deficiency in DNA non-homologous end-joining. DNA Repair 16, 84–96. 10.1016/j.dnarep.2014.02.011 24629483

[B231] Wu H. Thijssen P. E. de Klerk E. Vonk K. K. D. Wang J. den Hamer B. (2016). Converging disease genes in ICF syndrome: ZBTB24 controls expression of CDCA7 in mammals. Hum. Mol. Genet. 25, 4041–4051. 10.1093/hmg/ddw243 27466202

[B232] Wu H. Zeng H. Dong A. Li F. He H. Senisterra G. (2013). Structure of the catalytic domain of EZH2 reveals conformational plasticity in cofactor and substrate binding sites and explains oncogenic mutations. PLoS ONE 8, e83737. 10.1371/journal.pone.0083737 24367611 PMC3868588

[B233] Wu X. Zhang Y. (2017). TET-Mediated active DNA demethylation: Mechanism, function and beyond. Nat. Rev. Genet. 18, 517–534. 10.1038/nrg.2017.33 28555658

[B234] Xie M. Lu C. Wang J. McLellan M. D. Johnson K. J. Wendl M. C. (2014). Age-related mutations associated with clonal hematopoietic expansion and malignancies. Nat. Med. 20, 1472–1478. 10.1038/nm.3733 25326804 PMC4313872

[B235] Xu G.-L. Bestor T. H. Bourc’his D. Hsieh C.-L. Tommerup N. Bugge M. (1999). Chromosome instability and immunodeficiency syndrome caused by mutations in a DNA methyltransferase gene. Nature 402, 187–191. 10.1038/46052 10647011

[B236] Yu J.-R. Lee C.-H. Oksuz O. Stafford J. M. Reinberg D. (2019a). PRC2 is high maintenance. Genes Dev. 33, 903–935. 10.1101/gad.325050.119 31123062 PMC6672058

[B237] Yu X. Yang L. Li J. Li W. Li D. Wang R. (2019b). De novo and inherited SETD1A variants in early-onset epilepsy. Neurosci. Bull. 35, 1045–1057. 10.1007/s12264-019-00400-w 31197650 PMC6864154

[B238] Zemach A. McDaniel I. E. Silva P. Zilberman D. (2010). Genome-wide evolutionary analysis of eukaryotic DNA methylation. Science 328, 916–919. 10.1126/science.1186366 20395474

[B239] Zhao B. Madden J. A. Lin J. Berry G. T. Wojcik M. H. Zhao X. (2022). A neurodevelopmental disorder caused by a novel de novo SVA insertion in exon 13 of the SRCAP gene. Eur. J. Hum. Genet. 30, 1083–1087. 10.1038/s41431-022-01137-3 35768521 PMC9437004

[B240] Zheng S. C. Breeze C. E. Beck S. Teschendorff A. E. (2018). Identification of differentially methylated cell types in epigenome-wide association studies. Nat. Methods 15, 1059–1066. 10.1038/s41592-018-0213-x 30504870 PMC6277016

[B241] Zilberman D. Coleman-Derr D. Ballinger T. Henikoff S. (2008). Histone H2A.Z and DNA methylation are mutually antagonistic chromatin marks. Nature 456, 125–129. 10.1038/nature07324 18815594 PMC2877514

